# Galactic Circos: User-friendly Circos plots within the Galaxy platform

**DOI:** 10.1093/gigascience/giaa065

**Published:** 2020-06-12

**Authors:** Helena Rasche, Saskia Hiltemann

**Affiliations:** 1 Bioinformatics Group, Department of Computer Science, University of Freiburg, Georges-Köhler-Allee 106, 79110 Freiburg im Breisgau, Germany; 2 Erasmus Medical Center, Clinical Bioinformatics Group, Department of Pathology, Wytemaweg 80, 3015 CN, Rotterdam, The Netherlands

**Keywords:** genomics, visualization, Galaxy, Circos, UI/UX

## Abstract

**Background:**

Circos is a popular, highly flexible software package for the circular visualization of complex datasets. While especially popular in the field of genomic analysis, Circos enables interactive graphing of any analytical data, including alternative scientific domain data and non-scientific data. This high degree of flexibility also comes with a high degree of complexity, which may present an obstacle for researchers not trained in programming or the UNIX command line. The Galaxy platform provides a user-friendly browser-based graphical interface incorporating a broad range of “wrapped” command line tools to facilitate accessibility.

**Findings:**

We have developed a Galaxy wrapper for Circos, thus combining the power of Circos with the accessibility and ease of use of the Galaxy platform. The combination substantially simplifies the specification and configuration of Circos plots for end users while retaining the power to produce publication-quality visualizations of complex multidimensional datasets.

**Conclusions:**

Galactic Circos enables the creation of publication-ready Circos plots using only a web browser, via the Galaxy platform. Users may download the full set of Circos configuration files of their plots for further manual customization. This version of Circos is available as an open-source installable application from the Galaxy ToolShed, with its use clarified in a training manual hosted by the Galaxy Training Network.

## Findings

### Background

The Circos visualization tool [1] is widely used in the biological scientific community and is especially popular for use in scientific publications. Circos has >4,000 citations, and its plots have appeared on the cover of several leading scientific journals [2]. Its popularity is due in large part to its great flexibility; Circos offers a wide range of visualization options, and all aspects of a Circos plot may be customized to the user’s needs. While originally created for the visualization of genomic data, Circos makes no a priori assumptions about the format and domain of the input data; this is illustrated by the fact that it has been used for a wide range of applications, from genomics research to visualizations of car sales, urban planning, and presidential debates [3].

With Circos’s great flexibility also comes a high degree of complexity and a substantial learning curve, and as a result its use is often limited to expert users who are experienced with programming and the UNIX command line.

The Galaxy platform [4] aims to provide a user-friendly interface to command line tools and empower domain experts to run powerful analysis and visualization tools without the need for any programming experience. Galaxy offers a wide range of tools for a variety of applications domains and is widely used in the biological scientific community (≥8,900 citations, ≥7500 tools [5,6]). Galaxy also automates the installation of tools and all their dependencies, removing another hurdle for its use by research scientists.

Our tool combines the power of Circos with the user-friendliness of the Galaxy interface to greatly increase the accessibility of the tool and simplify the creation of publication-ready plots for scientific data.

Previously, custom Circos Galaxy plotter tools have been written [7]; however, these tools are not generic but are tailored specifically to the use-case at hand. This means that a new Galaxy tool has to be created whenever a new plot type is needed. Galactic Circos aims to be a generic tool capable of creating any Circos plot regardless of data domain.

### Results

**Figure 1:**
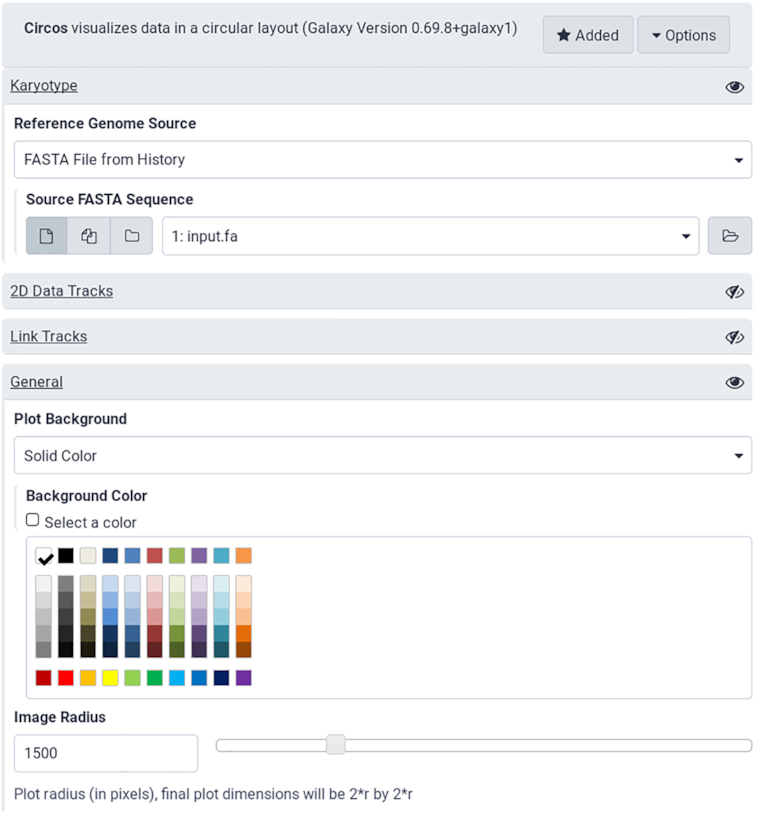
The Galaxy tool interface to Circos. Each collapsed section hides a wealth of configuration options available to users. The web-based interface is substantially more accessible than the command line version.

The Galactic Circos tool changes the way users must specify the configuration of a Circos plot. Instead of writing a number of configuration files, users now only need to select the various plot options from a web interface, and datasets from their analysis history (Fig. 1). Because Circos plot specifications can be quite complex, the tool interface is subdivided into several collapsible sections, each corresponding to a different Circos configuration option in order to increase the usability of the tool. Parameters are pre-configured with sensible default values so that basic plots can be generated with minimal configuration.

**Figure 2:**
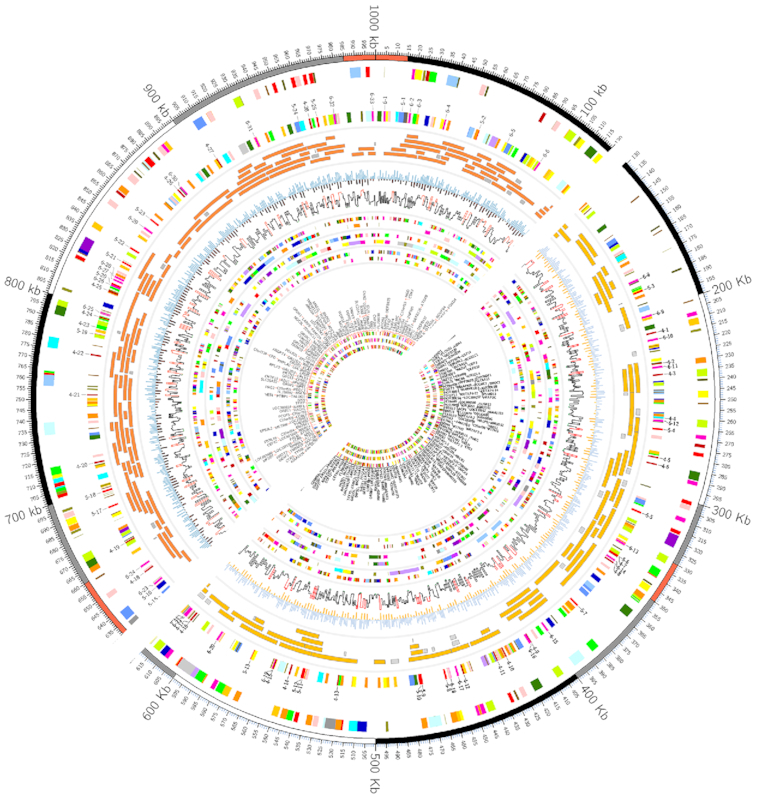
Here we reproduce one of the more complex tutorials from the Circos documentation. The upper left half of the image is produced by the configuration provided by the Circos tutorial, while the lower right half is produced completely in Galaxy. While some options used in the original tutorial cannot be directly used (e.g. unrestricted Perl code), they can be recreated equivalently in the tool interface. Some options in the tool interface are likewise restricted; Galactic Circos offers a color picker with a limited palette, which accounts for the differences in color. However, our tool offers the ability to download the full Circos configuration folder, allowing advanced users to configure the color (or other) parameters manually and rebuild the image locally. See https://usegalaxy.eu/u/helena-rasche/h/circos-microbe-tutorial.

We demonstrate the utility of the Galactic Circos tool by recreating one of the more advanced examples from the Circos online tutorials, the microbial genome lesson [8] (Fig. 2). This displays multiple tracks of different types (text, histogram, tiles), has a customized ideogram, and uses rules for coloring data points dependent on their value.

**Figure 3:**
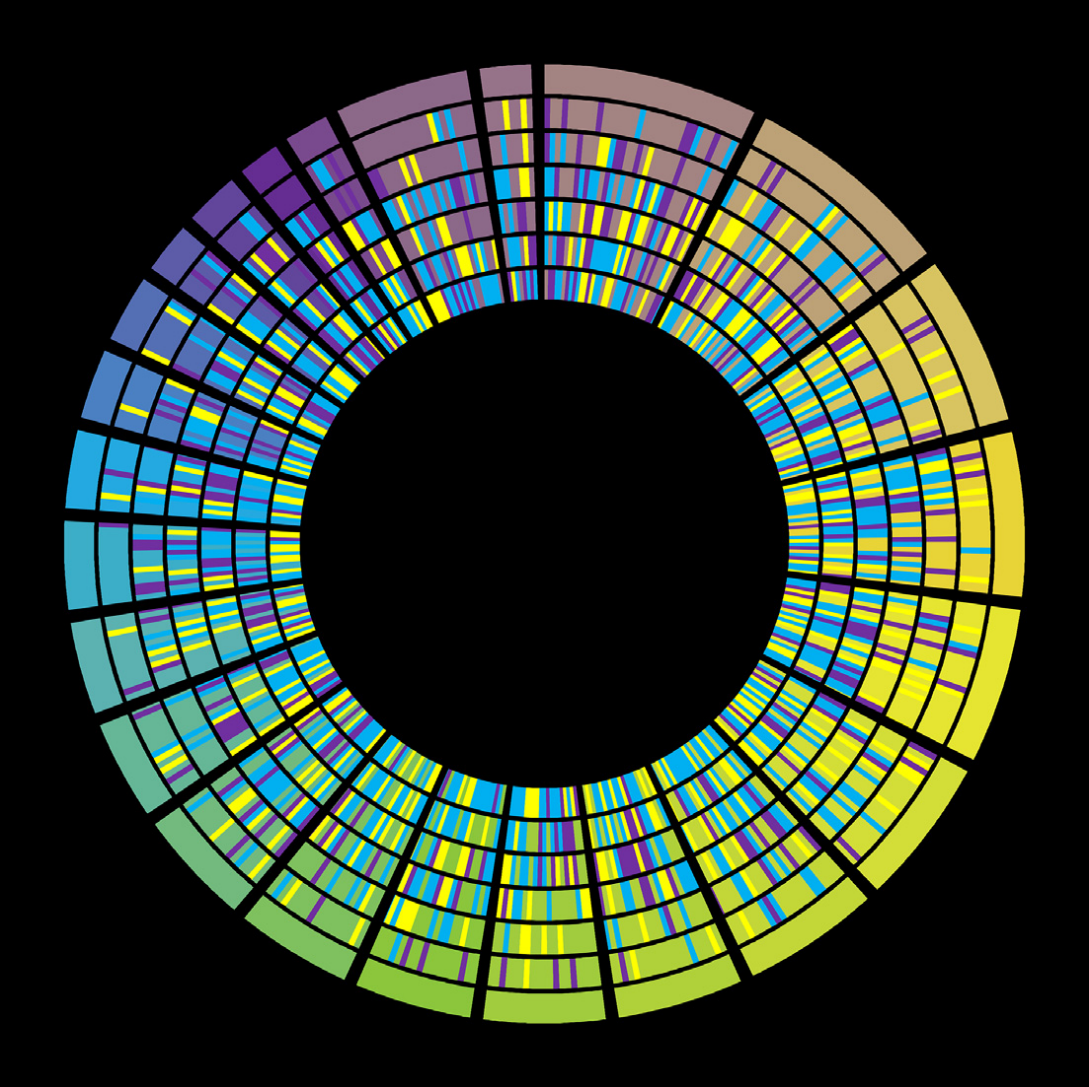
*Nature* cover [9] for the ENCODE project in September 2012, reproduced by Galactic Circos. The image is not a split image owing to copyright restrictions on the original cover image. Comparison can be made against the Circos tutorial [12]. https://usegalaxy.eu/u/helena-rasche/h/circos-encode-nature-cover.

In a second example (Fig. 3), we replicate within Galaxy the cover image of the *Nature* issue [9] dedicated to the ENCODE project [10]. This cover featured a Circos plot and is also available as part of the official Circos tutorials [11].

These 2 examples showcase a variety of different track types (histograms, scatterplot, highlights, tiles, text) and configurations (ticks, rules, ideogram customizations) to illustrate the feature-completeness of Galactic Circos.

#### Workflow summary

**Figure 4:**
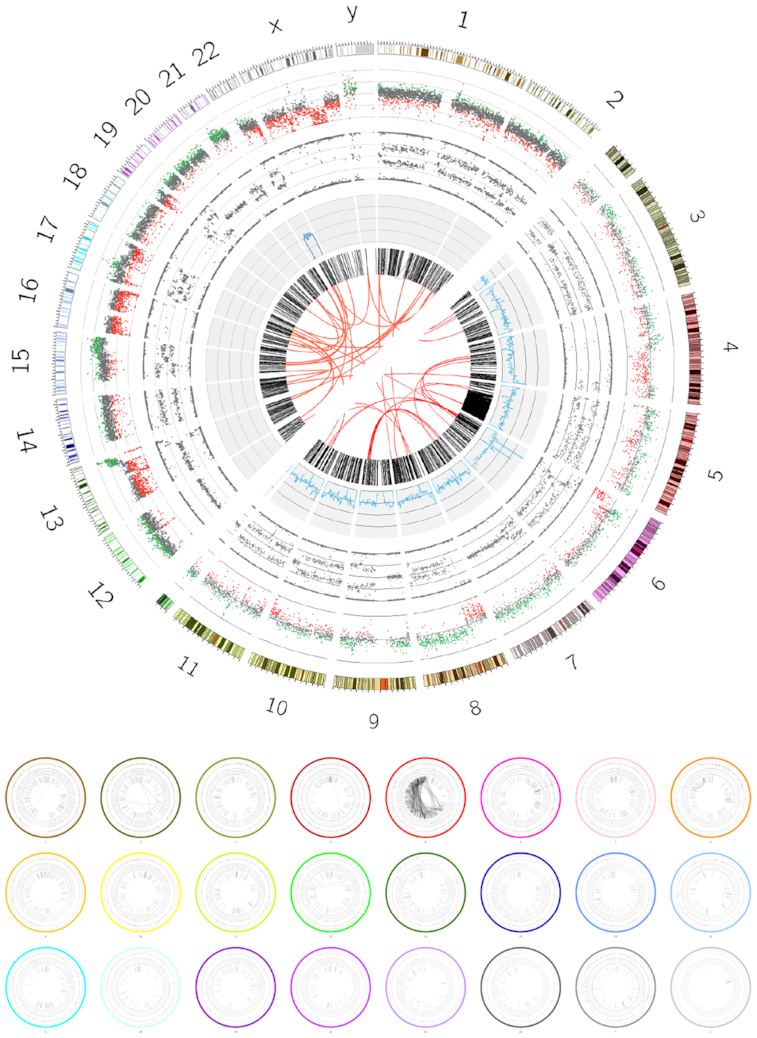
In the top panel, comparison of the output of a custom-written Circos plot with hard-coded configuration (upper left half) to the output created using the Galactic Circos tool (lower right half). While the input data originated from a range of standard and non-standard genomic file formats, conversion to Circos-formatted files was possible using the plethora of file manipulation tools already integrated into Galaxy and the set of supporting conversion tools included in the Galactic Circos package. In the bottom panel we produce Circos plots per chromosome, leveraging Galaxy’s ability to map a tool execution across a collection of input datasets, in this case each karyotype in a separate input file. The images are reduced and placed together in a montage using further Galaxy tools. https://usegalaxy.eu/u/helena-rasche/h/circos-cancer-genomics--chromothripsis, https://usegalaxy.eu/u/helena-rasche/h/circos-multiplot.

Visualizations in the Galaxy framework are usually implemented as interactive JavaScript components, but these plots cannot be created automatically in workflows. Individual plotting tools exist as Galaxy tools; however, these are less common and generally less flexible because tool authors must make a trade-off between development time and feature support. We put significant time into the development in order to make an extremely generic tool, enabling researchers to use the Galactic Circos tool in their workflows, based on previous experiences building single-purpose Circos plotting tools (e.g., as in Fig. 4). This enables creation of human-readable summaries of large analysis workflows, similar to the non-genomics–focused iReport [13]. Galactic Circos was born from precisely this use-case and therefore aims to enable reducing complex analysis pipeline outputs, such as the workflows required in cancer genomics, allowing bioinformaticians to produce a single image summarizing all of their relevant outputs in an easily digestible manner.

#### Supporting tools

Circos requires input datasets to adhere to a specific and custom file format. To facilitate the conversion of data to this custom Circos format, we have developed several supporting Galaxy tools for conversion. These tools allow users to convert their datasets from a variety of common genomics formats such as (big)Wig files, interval files, and MAF/Stockholm alignments. Furthermore, the existing Galaxy ecosystem provides a wide array of tabular data manipulation tools that can be leveraged to transform any tabular or text files into the format accepted by Circos.

To demonstrate the utility of these supporting tools, we show a real-world example of a plot using common genomics datasets. This example is a re-creation of a plot in a published article demonstrating chromothripsis in the VCaP prostate cancer cell line [14]. The input datasets originate from a variety of sources, including a structural variants files (converted to Circos links track), copy number and B-allele frequency track obtained from Affymetrix single-nucleotide polymorphism (SNP) array data, and a SNP density track generated from a VCF file. Using a combination of the supporting tools included in the Galactic Circos package and the generic file manipulation tools present in Galaxy, we were able to convert these various datasets to Circos-compatible formats without leaving Galaxy, and reproduced the Circos plot from the publication (Fig. 4).

**Figure 5:**
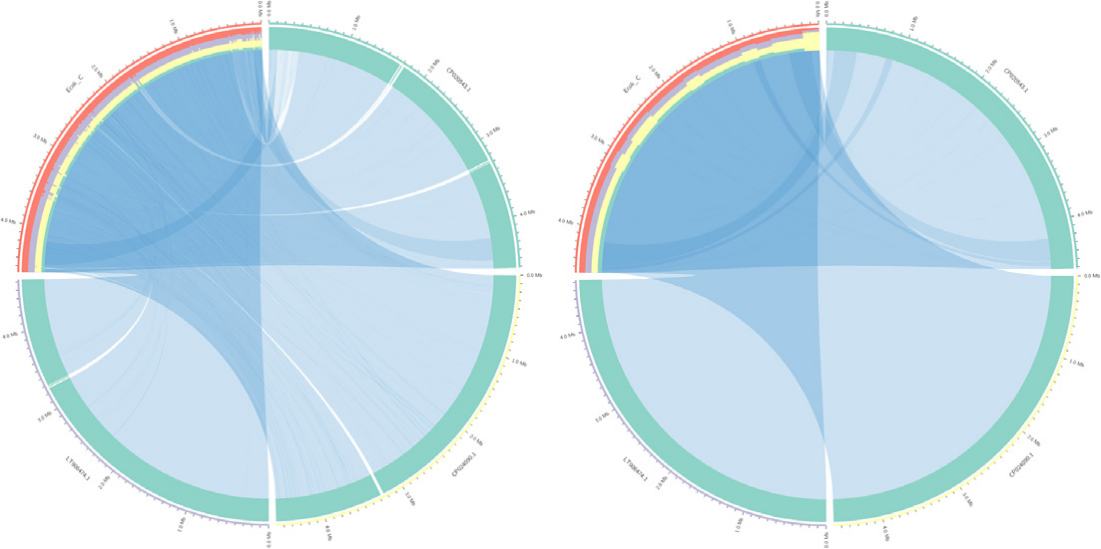
These 2 plots show the link-binning and bundling scripts used with different thresholds. The inner link track was generated directly from a MAF file output by LastZ [15]. This file was processed by Circos’s bundling tool in Galaxy to decrease the number of links, a process usually done to decrease visual noise and increase plotting efficiency. The outer track demonstrates the link-binning script, which generates a histogram, in this case from the number of links to that position in the genomic region.

Once data have been reformatted for Circos, they can either be used immediately or be further processed. Circos includes a tool suite for post-processing and down-sampling of data, which can improve plot clarity and processing speed. We additionally included a number of these post-processing tools into Galaxy, notably the link-bundling and binning tools used in Fig. 5.

**Figure 6:**
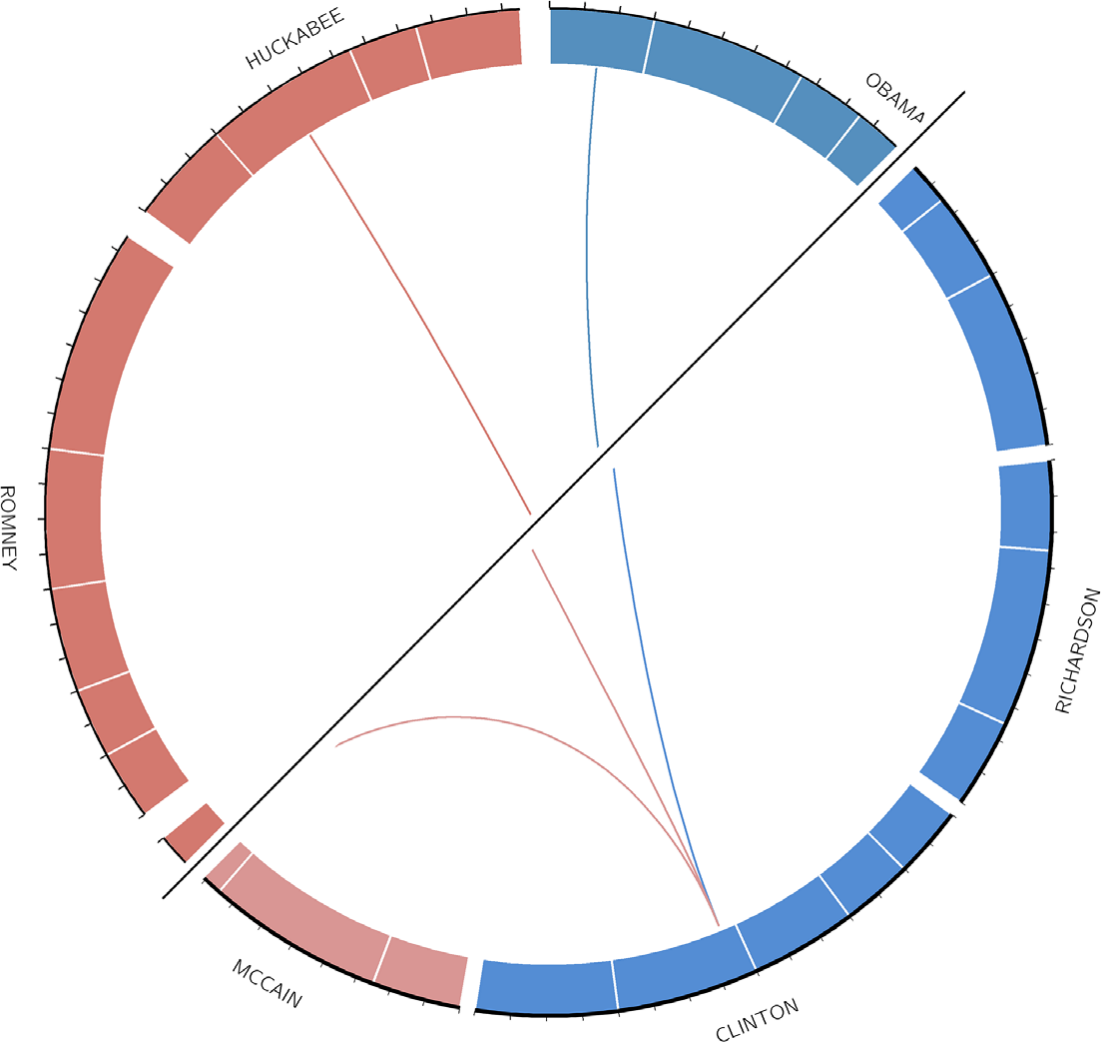
This figure compares the Circos plot from the official tutorial (upper left half) to the output created using the Galactic Circos tool (lower right half). Each link represents a candidate speaking the last name of another candidate. The length of each circle segment is proportional to the total number of words spoken by the candidate during the debates. https://usegalaxy.eu/u/saskia/h/circos-politics-plot.

Finally, while Circos is widely used for the visualization of genomic data, and many of the parameter names have a distinctly biological feel to them, the tool does not impose any restrictions on the type of input data and is capable of displaying non-biological data just as easily [3]. To show that our tool retains this degree of flexibly, we recreated the presidential debate plot included in the Circos tutorials, which in turn was based on a plot that appeared in a *New York Times* article [16]. A plot comparison can be seen in Fig. 6.

### Lessons learned and limitations

Given the great flexibility and configurability of the Circos tool, our Galaxy wrapper is, to our knowledge, one of the most complex Galaxy tools. Development of this wrapper took significant time and resources, and in places took us to the edges of what is possible in Galaxy. In this section we describe some of the lessons learned and tips for wrapping tools of this complexity.

#### Security

This wrapper exposes ∼95% of what is possible with Circos. We intentionally excluded the last ∼5% of features because we could not safely implement them. These features would require allowing free-text user input and unrestricted Perl code, which can pose a potential security risk. We believed that we could not, within a reasonable period of development time, implement sufficient sanitization of all possible user inputs. Instead, we provide an option for the tool to output the full set of configuration files required to recreate the plot, which the user can use as a starting point for manual adaptation locally. There are ongoing efforts within the Galaxy community to perform computations with increasingly untrusted user input, and we hope that the Galaxy community will push this even further in the future and make it the default, rather than requiring special configuration and knowledge from system administrators. This would enable us to add a free-text field within the Circos tool, and users could provide custom configuration freely and without risk to the administrator.

#### Visualization vs tool

We made the initial choice to build Galactic Circos as a tool, not a visualization, given the long compilation times of plots and our desire to build a workflow-compatible tool because this was not possible in Galaxy at that time. In the future, we might want to explore the possibilities of a more dynamic visual interface, using a visualization plugin in Galaxy. We would have complete freedom to build in more interactivity and custom components (e.g., visual preview for Brewer scale selection) as needed.

#### Macros

Macros proved incredibly helpful in wrangling the complexity of this tool by allowing us to define reusable components and avoid code duplication. Galaxy wrappers allow for the definition of "macros"; these are bits of code defined in a file outside the main wrapper and can be reused at multiple points in the tool. Unfortunately, the extent to which this tool relies on macros also makes the tool more complex from a development point of view, with the code spread out over a large number of files. However, the benefits here outweigh the drawbacks.

#### Collapsible sections

The section feature in Galaxy permits grouping related options together in the user interface. This avoids overwhelming the user with the enormous array of available parameters but rather groups these logically and only shows those subsets requested by the user. Unfortunately, these sections re-collapse themselves during tool rerun and are not marked when their children contain modifications from the defaults. If this was changed, users could more easily recall what they did in the previous tool run because all edited sections would be expanded, or marked by default.

#### Color

The built in color selector provides a small palette of colors. While it is a good thing to prevent users from making plots with hard-to-see or unpleasant colors, it also substantially limits more advanced users. The addition of an advanced color picker would be welcome for Circos users. Likewise we used a select box for Brewer palette, which feels suboptimal compared to a component that could include a preview of that palette and would be much more user-friendly.

## Methods

### Implementation

The execution of the tool leverages Galaxy’s ability to write templated files directly to disk with configuration from the tool form, and then run Circos directly on these templated configuration files.

Installation of the Circos tool and its dependencies is handled by the Galaxy platform, which supports different dependency management frameworks, including Conda and Containers. All dependencies including Circos itself are available from the Bioconda Conda channel [17] and available as a virtualized container (rkt, Docker, Singularity). The version of the Galaxy Circos tool being reported on here uses Circos version 0.69.8.

### File format converters

To facilitate interoperability with upstream tools and workflows, we provide a set of file format converters, in addition to many tools already available in Galaxy, which together provide for conversion of a range of common data format standards (e.g., VCF, MAF/Stockholm, BED/GFF3, BigWig). These tools produce files that are ready to be used as input to the Galaxy Circos tool. Additionally the applicable subset of circos-utils were included into Galaxy for Circos-friendly tools for data reshaping.

### Circos configuration export

While Galactic Circos aims to offer the full range of Circos functionality, some manual customization of the Circos plot configuration files may still be desired. To this end, our tool also outputs the full set of configuration files needed to recreate the plot on the command line and thus allow easy access to any features not exposed in the Galaxy wrapper.

### Training materials

Our tool greatly simplifies the creation of Circos plots, but the large number of options offered by the Circos tool necessitates good documentation and explanation to optimize their utility for end-users. Circos offers a collection of tutorials that are designed to familiarize users with the various features of Circos [18]. In a similar fashion, we have created a set of Galaxy tutorials aimed to educate users in the use of Circos within Galaxy. These tutorials are available from the Galaxy training materials website [19].

### Reproducible and reusable plots

To enable readers to examine the complete parameter settings used and recreate the example plots given here, Galaxy histories for all the figures shown in this work have been made publicly available from the European Galaxy server (see Availability section).

### Future work

While we have aimed to make our tool as feature-complete as possible, some of Circos’s functionality is not currently exposed in the Galaxy tool. We intend to extend our tool to include these features, including but not limited to support for scaling subsections of the plots, and generation of HTML image maps.

## Availability of Source Code and Requirements

- Project name: Galactic Circos
- bio.tools ID: galactic_circos
- RRID:SCR_018207
- Github repository: https://github.com/galaxyproject/tools-iuc/tree/master/tools/circos
- ToolShed repository: https://toolshed.g2.bx.psu.edu/view/iuc/circos
- Training Manual: https://training.galaxyproject.org/training-material/topics/visualisation/tutorials/circos/tutorial.html
- Operating system(s): Unix (Platform independent with Docker, Singularity)
- Other requirements: Galaxy version 18.01 or higher
- License: MIT

The Circos example plots presented in this work are available as Galaxy histories:

- Galaxy history for Figure 2: https://usegalaxy.eu/u/helena-rasche/h/circos-microbe-tutorial
- Galaxy history for Figure 3: https://usegalaxy.eu/u/helena-rasche/h/circos-encode-nature-cover
- Galaxy history for Figure 4a: https://usegalaxy.eu/u/helena-rasche/h/circos-cancer-genomics--chromothripsis
- Galaxy history for Figure 4b: https://usegalaxy.eu/u/helena-rasche/h/circos-multiplot
- Galaxy history for Figure 6: https://usegalaxy.eu/u/saskia/h/circos-politics-plot

**Galaxy Resources**

- Galaxy Home Page: https://galaxyproject.org
- Galaxy Tutorials: https://training.galaxyproject.org
- How to install Galaxy: https://getgalaxy.org
- How to install tools: https://galaxyproject.org/admin/tools/add-tool-from-toolshed-tutorial/
- Full Administrative resources: https://docs.galaxyproject.org
- Galaxy Help Forum: https://help.galaxyproject.org
- Connect with the Galaxy Community on Gitter Chat: https://gitter.im/galaxyproject/Lobby/
- Public Galaxy servers that include Circos: usegalaxy.eu, usegalaxy.org, usegalaxy.org.au (see Galactic Circos tutorial for full up-to-date list)

## Availability of Supporting Data and Materials

The data presented here to illustrate our application were obtained from previous publications and have been collected and made available from Zenodo [20].

Additional supporting data are available from the GigaScience GigaDB database [21].

## Abbreviations

BED: Browser Extensible Data; ENCODE: Encyclopedia of DNA Elements; GFF: general feature format; HTML: HyperText Markup Language; MAF: multiple alignment format; SNP: single-nucleotide polymorphism; VCF: variant call format.

## Competing Interests

The authors declare that they have no competing interests.

## Funding

This project was made possible with the support of the Albert Ludwig University of Freiburg and German Federal Ministry of Education and Research (031 L0101C de.NBI-epi).

Funding for open access charge: German Federal Ministry of Education and Research.

This project has received funding from the European Union’s Horizon 2020 research and innovation program under grant agreement 825775.

## Authors' Contributions

H.R. and S.H. contributed equally to the tool development, documentation, and writing of the manuscript.

## Supplementary Material

giaa065_GIGA-D-20-00031

giaa065_GIGA-D-20-00031_R1

giaa065_GIGA-D-20-00031_R2

giaa065_Response_to_Reviewer_Comments_Original_Submission

giaa065_Response_to_Reviewer_Comments_Revision_1

giaa065_Reviewer_1_Report_Original_Submission Martin Krzywinski, M.Sc. -- 2/20/2020 Reviewed

giaa065_Reviewer_1_Report_Revision_1 Martin Krzywinski, M.Sc. -- 5/11/2020 Reviewed

giaa065_Reviewer_2_Report_Original_Submission Jennifer Hillman-Jackson -- 2/25/2020 Reviewed

giaa065_Reviewer_3_Report_Original_Submission Jeremy Goecks -- 2/26/2020 Reviewed
