# Supplementary material for: Galactic Circos: User-friendly Circos plots within the Galaxy platform
Source: Gigascience. 2020 Jun 12;9(6):giaa065. doi: 10.1093/gigascience/giaa065 (PMC7291503; doi:10.1093/gigascience/giaa065)
Supplement: giaa065_GIGA-D-20-00031 [file giaa065_giga-d-20-00031.pdf]

GigaScience

Galactic Circos: User friendly Circos Plots within the Galaxy platform

--Manuscript Draft--

**Manuscript Number:**	GIGA-D-20-00031
**Full Title:**	Galactic Circos: User friendly Circos Plots within the Galaxy platform
**Article Type:**	Technical Note
**Funding Information:**	German Federal Ministry of Education and Research (031 L0101C de.NBI-epi) — Ms. Helena Rasche<br>European Union Horizon 2020 (825775) — Ms. Saskia Hiltemann
**Abstract:**	Background: Circos is a popular software package for circular visualisation of complex datasets. Circos is a highly flexible tool and is especially popular in the field of genomics, though it may be applied to any type of data. This high degree of flexibility also comes with a high degree of complexity, which may present an obstacle for researchers not trained in programming or the UNIX command line. Galaxy provides a user-friendly graphical interface for such commandline tools, greatly improving their accessibility.<br>Findings: We have developed a Galaxy wrapper for Circos, thus combining the power of Circos with the accessibility and ease-of-use of the Galaxy platform. This significantly simplifies the specification of Circos plots for end-users while retaining the power to produce publication-quality visualization of multidimensional complex datasets.<br>Conclusions: Galactic Circos enables the creation of publication-ready Circos plots within the Galaxy platform. Users may download the full configuration of their plots for further manual tweaking. This tool has been made available from the Galaxy Tool Shed, and is accompanied by a training manual in the Galaxy Training Network.
**Corresponding Author:**	Helena Rasche<br>Bioinformatics Group, University of Freiburg<br>Freiburg, Baden-Württemberg GERMANY
**Corresponding Author Secondary Information:**	
**Corresponding Author's Institution:**	Bioinformatics Group, University of Freiburg
**Corresponding Author's Secondary Institution:**	
**First Author:**	Helena Rasche
**First Author Secondary Information:**	
**Order of Authors:**	Helena Rasche<br>Saskia Hiltemann
**Order of Authors Secondary Information:**	
**Additional Information:**	
**Question**	**Response**
Are you submitting this manuscript to a special series or article collection?	No
**Experimental design and statistics**<br><br>Full details of the experimental design and statistical methods used should be given in the Methods section, as detailed in our	No

Powered by Editorial Manager® and ProduXion Manager® from Aries Systems Corporation

Minimum Standards Reporting Checklist. Information essential to interpreting the data presented should be made available in the figure legends.<br><br>Have you included all the information requested in your manuscript?	
If not, please give reasons for any omissions below.<br><br>as follow-up to "**Experimental design and statistics**<br><br>Full details of the experimental design and statistical methods used should be given in the Methods section, as detailed in our Minimum Standards Reporting Checklist. Information essential to interpreting the data presented should be made available in the figure legends.<br><br>Have you included all the information requested in your manuscript?<br><br>"	No experiment was conducted, we simply report the production of a new piece of software? But if there is more information we can include we would be happy to
**Resources**<br><br>A description of all resources used, including antibodies, cell lines, animals and software tools, with enough information to allow them to be uniquely identified, should be included in the Methods section. Authors are strongly encouraged to cite Research Resource Identifiers (RRIDs) for antibodies, model organisms and tools, where possible.<br><br>Have you included the information requested as detailed in our Minimum Standards Reporting Checklist?	Yes
**Availability of data and materials**	Yes

All datasets and code on which the conclusions of the paper rely must be either included in your submission or deposited in publicly available repositories (where available and ethically appropriate), referencing such data using a unique identifier in the references and in the “Availability of Data and Materials” section of your manuscript.<br><br>Have you have met the above requirement as detailed in our Minimum Standards Reporting Checklist?	

Powered by Editorial Manager® and ProduXion Manager® from Aries Systems Corporation

Click here to access/download;LaTeX - Figure (eps, ps, etc.);plot-complete-genomics-multi.png

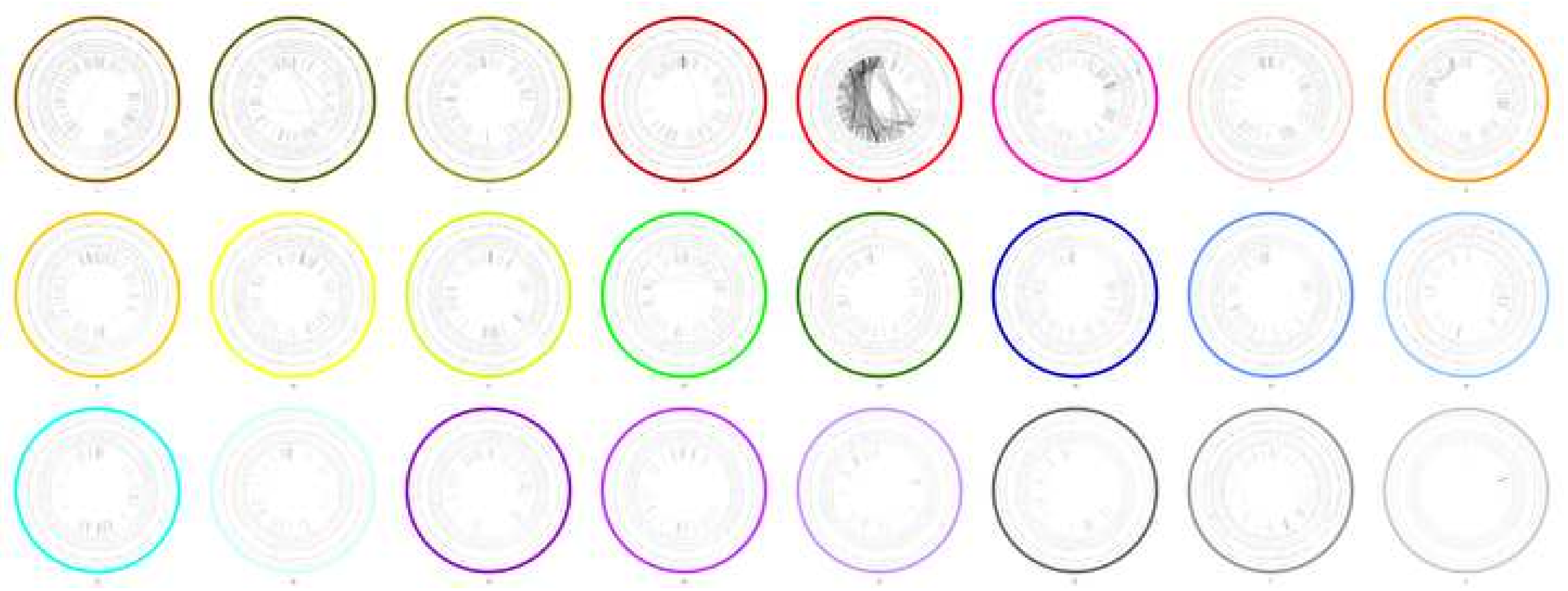

Click here to access/download;LaTeX - Figure (eps, ps, etc.);plot-politics-both.png

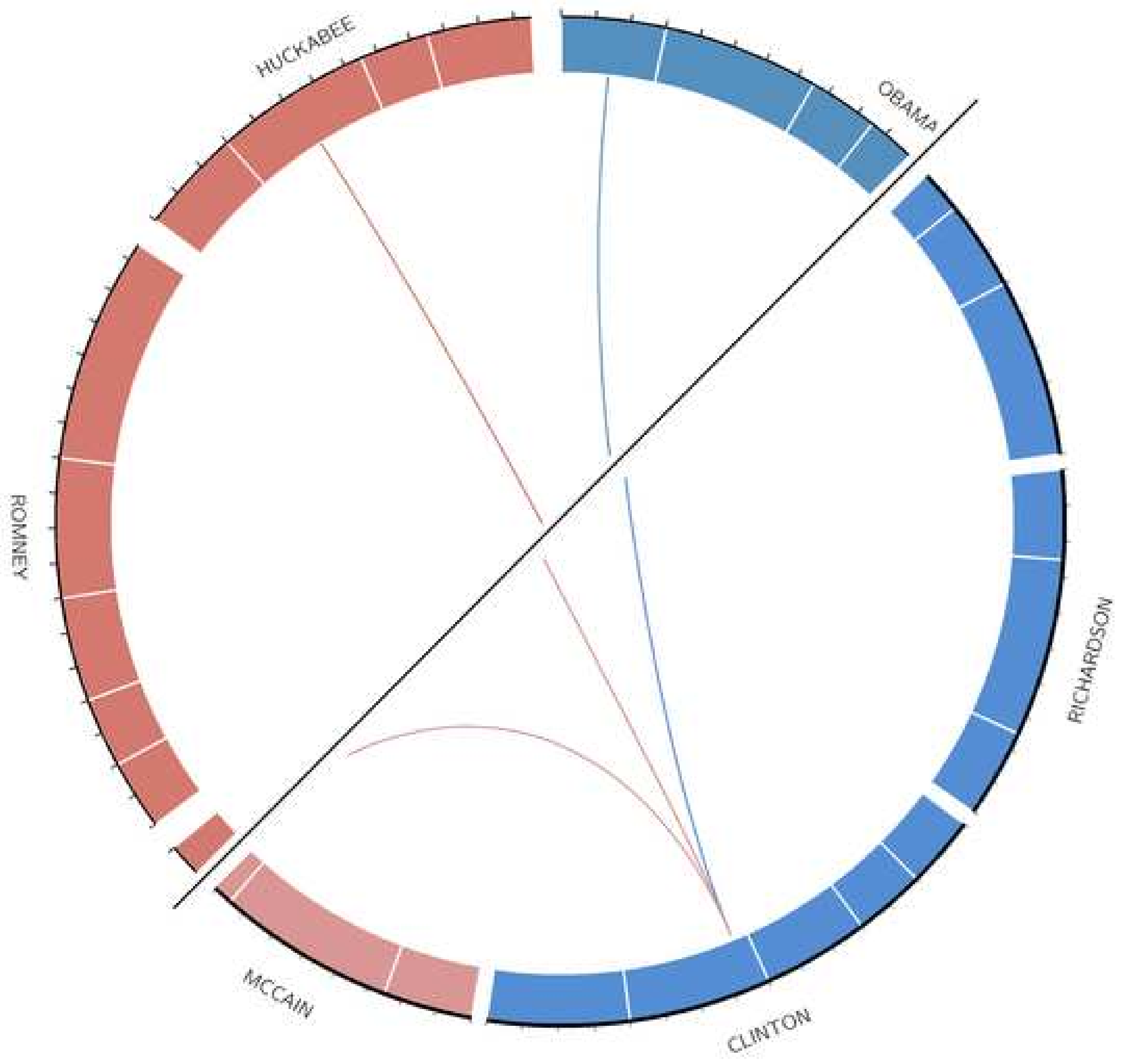

Click here to access/download;LaTeX - Figure (eps, ps, etc.);plot-encode.png

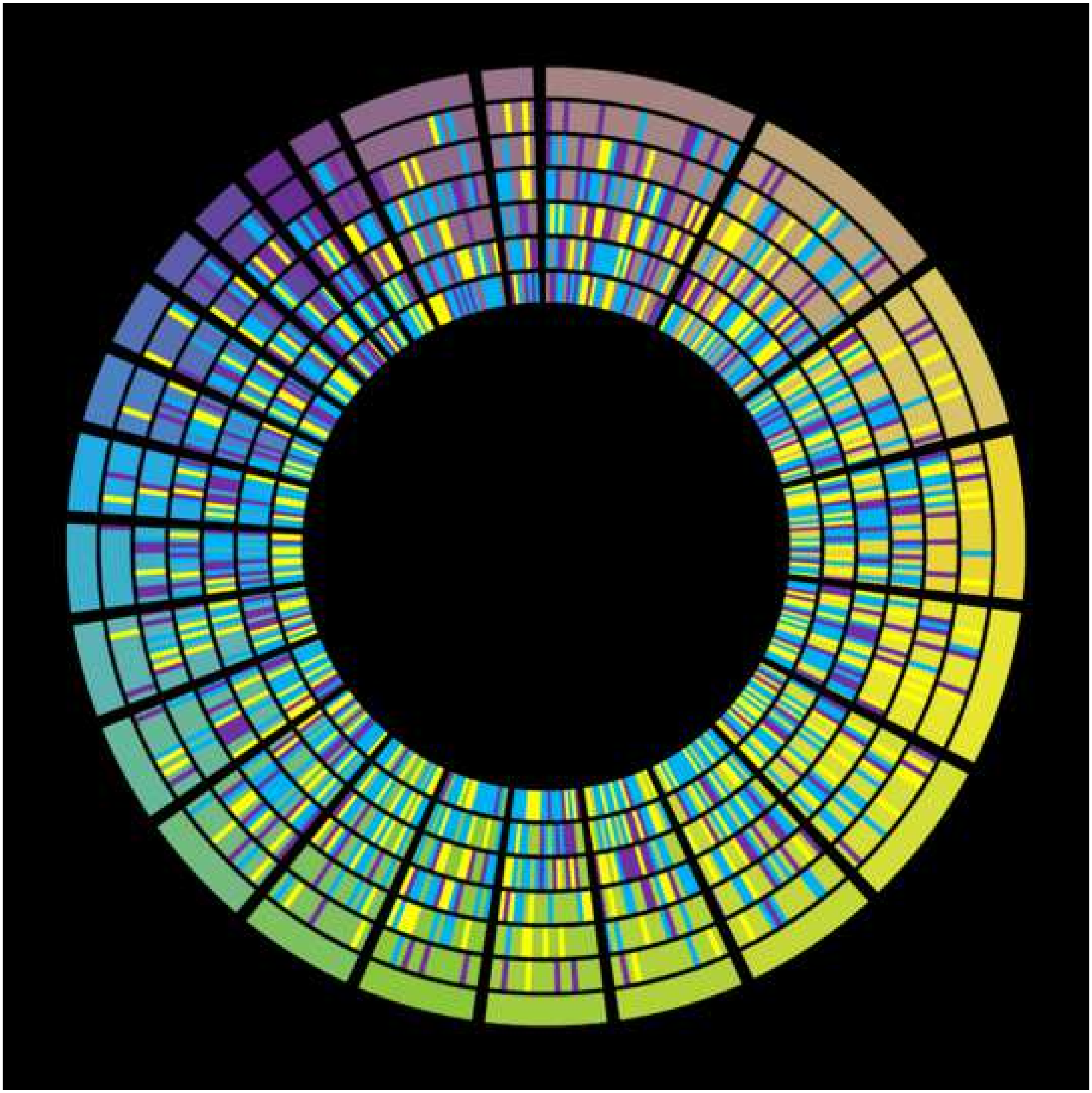

Click here to access/download;LaTeX - Figure (eps, ps, etc.);circos-galaxy-ui.png

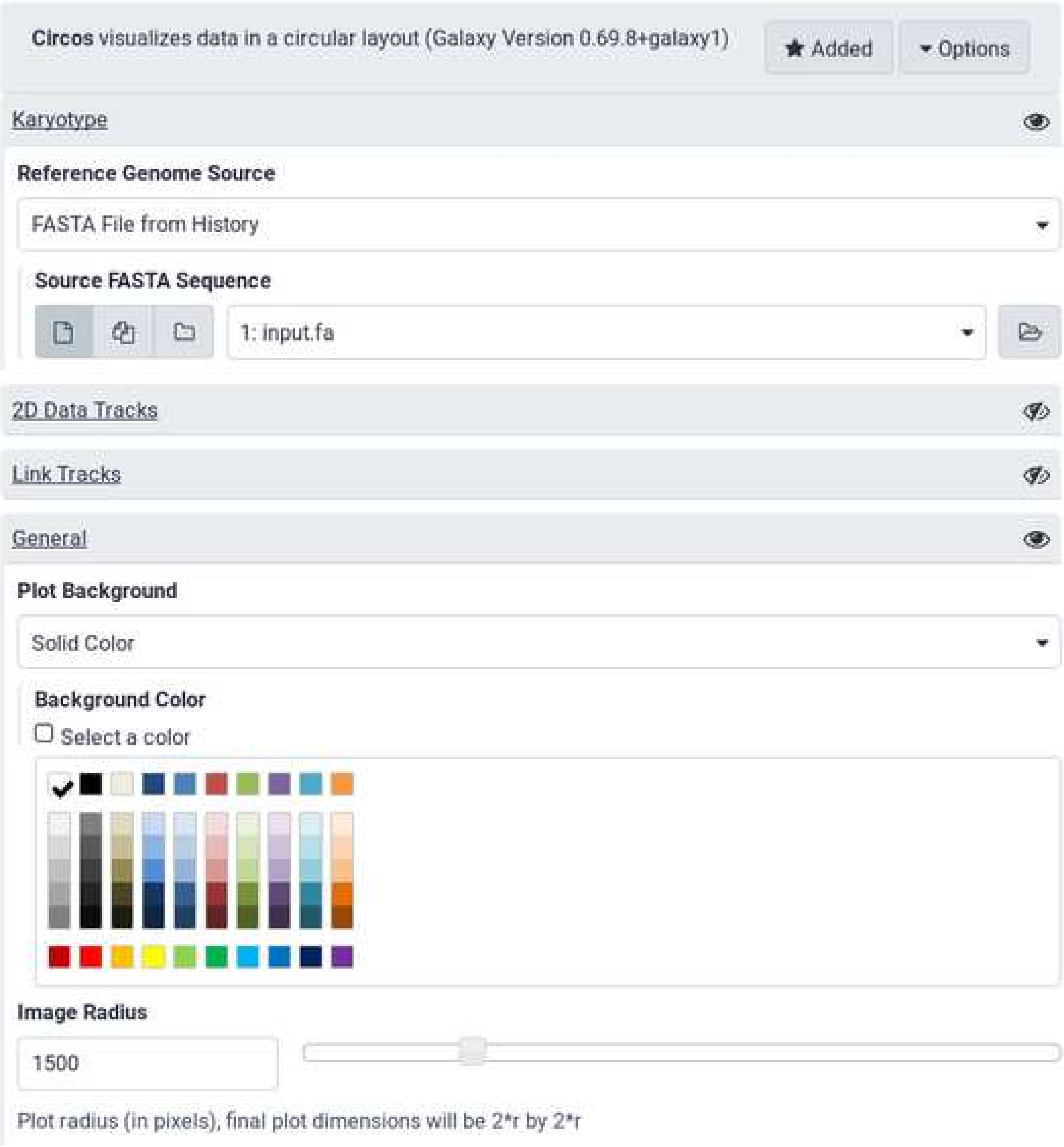

Click here to access/download;LaTeX - Figure (eps, ps, etc.);plot-complete-genomics-both.png

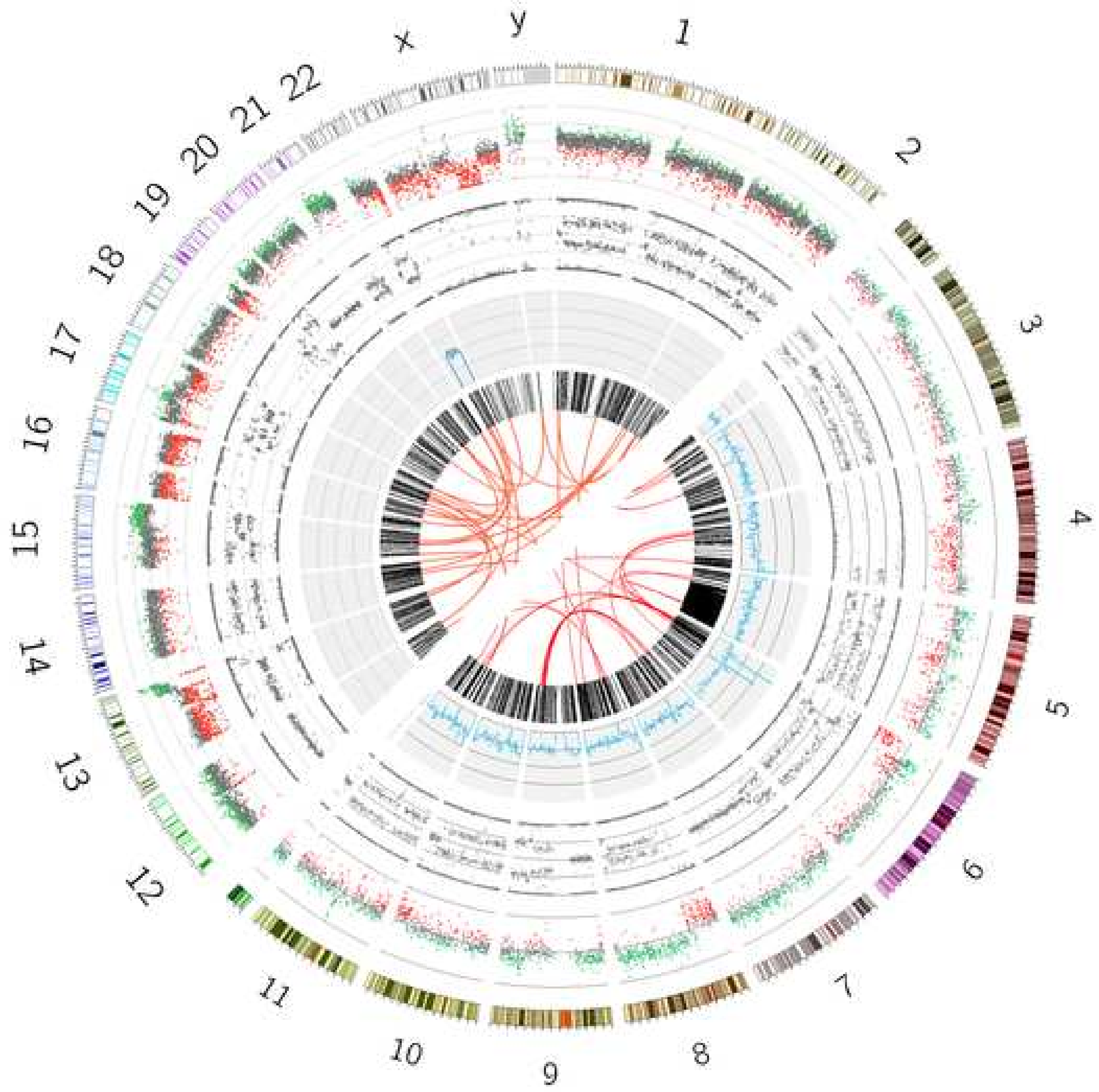

Click here to access/download;LaTeX - Figure (eps, ps, etc.);plot-microbe-both.png

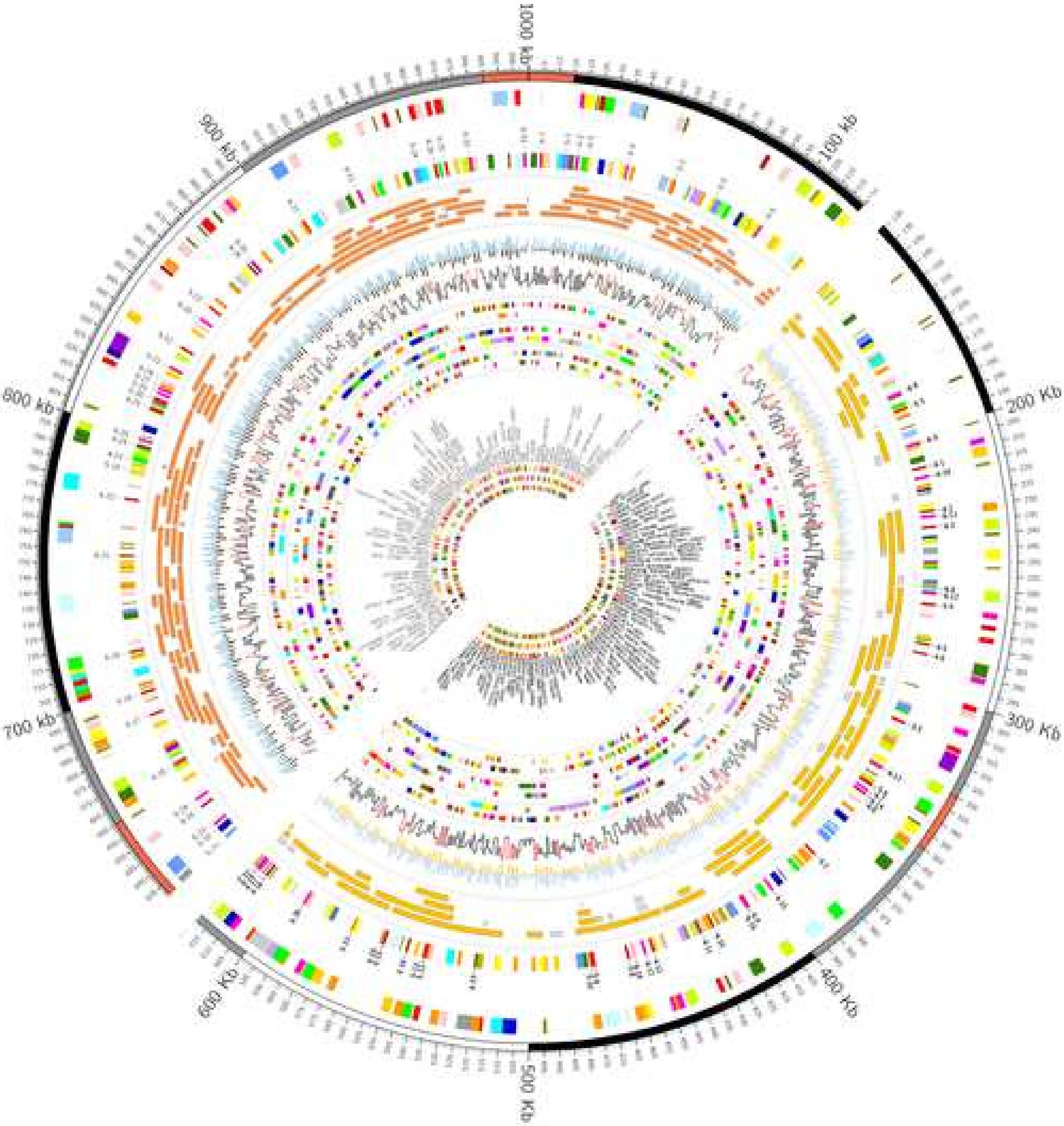

Manuscript
Click here to access/download;Manuscript;main.pdf

OXFORD
(GIGA)n SCIENCE

GigaScience, 2017, 1–5

doi: xx.xxxx/xxxx
Manuscript in Preparation
Technical Note

TECHNICAL NOTE

Galactic Circos: User friendly Circos Plots within the Galaxy platform

Helena Rasche[1,*,†] and Saskia Hiltemann[2,*,†]

[1]Bioinformatics Group, Department of Computer Science, University of Freiburg, 79110 Freiburg im Breisgau, Germany and [2]Erasmus Medical Center, Clinical Bioinformatics group, Department of Pathology, Wytemaweg 80, 3015 CN, Rotterdam, The Netherlands

*hxr@informatik.uni-freiburg.de; saskiahiltemann@gmail.com
†Contributed equally.

Abstract

Background: Circos is a popular software package for circular visualisation of complex datasets. Circos is a highly flexible tool and is especially popular in the field of genomics, though it may be applied to any type of data. This high degree of flexibility also comes with a high degree of complexity, which may present an obstacle for researchers not trained in programming or the UNIX command line. Galaxy provides a user-friendly graphical interface for such commandline tools, greatly improving their accessibility.
Findings: We have developed a Galaxy wrapper for Circos, thus combining the power of Circos with the accessibility and ease-of-use of the Galaxy platform. This significantly simplifies the specification of Circos plots for end-users while retaining the power to produce publication-quality visualization of multidimensional complex datasets.
Conclusions: Galactic Circos enables the creation of publication-ready Circos plots within the Galaxy platform. Users may download the full configuration of their plots for further manual tweaking. This tool has been made available from the Galaxy Tool Shed, and is accompanied by a training manual in the Galaxy Training Network.

Key words: Genomics; Visualisation; Galaxy; Circos; UI/UX

Findings

Background

The Circos visualization tool [19] is widely used in the biological scientific community, and is especially popular for use in scientific publications. Circos has over 4000 citations, and its plots have appeared on the cover of several leading scientific journals [8]. Its popularity is due in a large part to its great flexibility; Circos offers a wide range of visualisation options, and all aspects of a Circos plot may be tweaked and customized to the user's wishes. While originally created for the visualisation of genomic data, Circos makes no a priori assumptions about the format and domain of the input data; this is illustrated by the fact that it has been used for a wide range of applications, ranging from genomics research to visualisations of car sales, urban planning, and even presidential debates [9].

With Circos's great flexibility also comes a high degree of complexity, and a significant learning curve, and as a result its use is often limited to expert users who are experienced with programming and the UNIX command line.

The Galaxy platform [11] aims to provide a user-friendly interface to commandline tools, and empower domain experts to run powerful analysis and visualization tools without the need for any programming experience. Galaxy offers a wide range of tools for a variety of applications domains, and is widely used in the biological scientific community (8900+ citations, 7500+ tools [10, 5]). Galaxy also automates the installation of tools and all their dependencies, removing another hurdle for its use by research scientists.

Compiled on: January 31, 2020.
Draft manuscript prepared by the author.

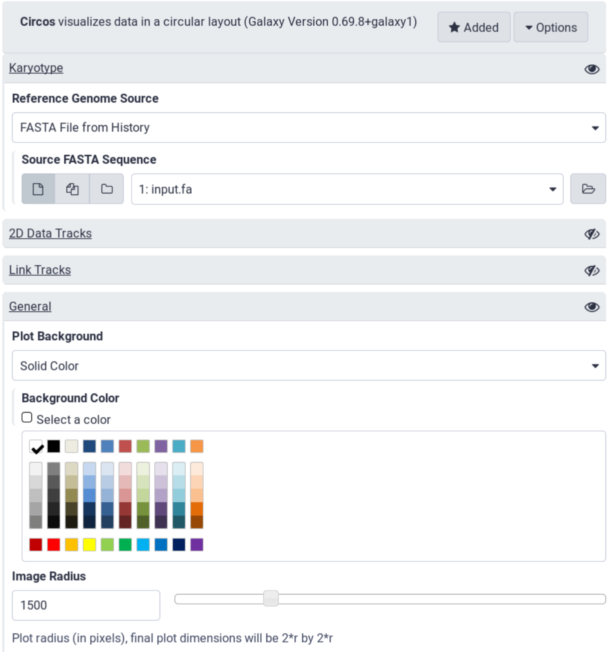

Figure 1. The Galaxy tool interface to Circos. Each collapsed section hides a wealth of configuration options available to users. The web based interface is significantly more accessible than the command line version.

Our tool combines the power of Circos with the user-friendliness of the Galaxy interface to greatly increase the accessibility of the tool and simplify the creation of publication-ready plots for scientific data.

Previously, custom Circos Galaxy plotter tools have been written [18]; however, these tools are not generic, but are tailored specifically to the use case at hand. This means that a new Galaxy tool has to be created whenever a new plot type is needed. Galactic Circos aims to be a generic tool capable of creating any Circos plot regardless of data domain.

Results

The Galactic Circos tool changes the way users must specify the configuration of a Circos plot. Instead of writing a number of configuration files, users now only need to select the various plot options from a web interface, and datasets from their analysis history (Figure 1). Because Circos plot specifications can be quite complex, the tool interface is subdivided into several collapsible sections, each corresponding to a different Circos configuration option in order to increase the usability of the tool. Parameters are preconfigured with sensible default values so that basic plots can be generated with minimal configuration.

We demonstrate the utility of the Galactic Circos tool by recreating one of the more advanced examples from the Circos online tutorials, the microbial genome lesson [2] (Figure 2). This displays multiple tracks of different types (text, histogram, tiles), has a customized ideogram, and uses rules for colouring data points dependent on their value.

In a second example (Figure 3), we replicate within Galaxy the cover image of the Nature issue [7] dedicated to the ENCODE project [14]. This cover featured a Circos plot and is also available as part of the official Circos tutorials [1].

These two examples showcase a variety of different track types (histograms, scatterplot, highlights, tiles, text) and configurations (ticks, rules, ideogram customizations) to illustrate the feature-completeness of Galactic Circos.

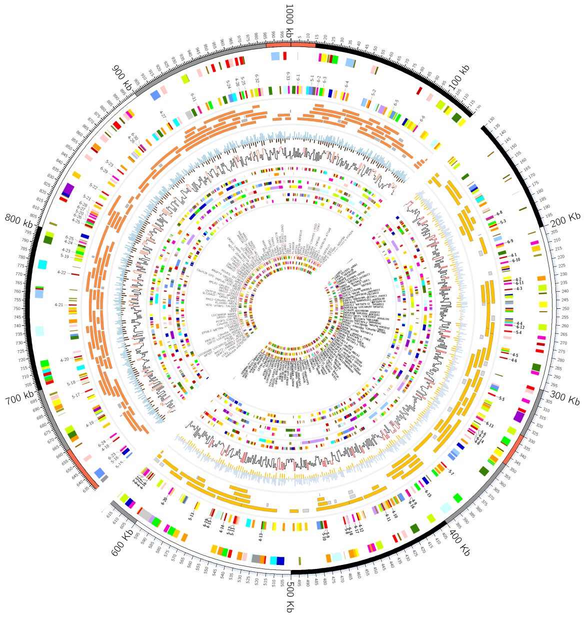

Figure 2. Here we reproduce one of the more complex tutorials from the Circos documentation. The top-left half of the image is produced by the configuration provided by the Circos tutorial, while the bottom-right half is produced completely in Galaxy. While some options used in the original tutorial cannot be directly used (e.g. unrestricted perl code), they can be recreated equivalently in the tool interface. Some options in the tool interface are likewise restricted, Galactic Circos offers a color picker with a limited palette, which explains the differences in colour. However, our tool offers the ability to download the full Circos configuration folder, allowing advanced users to tweak the colour (or other) parameters manually and rebuild the image locally. https://usegalaxy.eu/u/helena-rasche/h/circos-microbe-tutorial

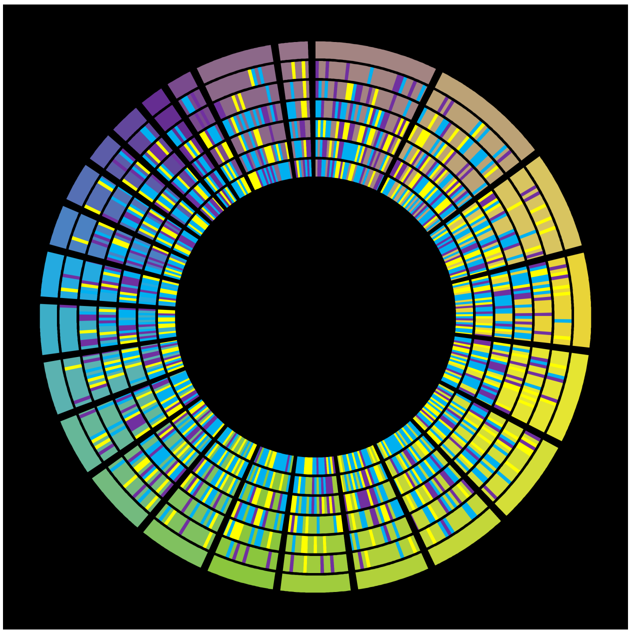

Figure 3. Nature's cover [7] for the ENCODE project in September 2012, reproduced by Galactic Circos. The image is not a split image due to copyright restrictions on the original cover image. Comparison can be made against the Circos tutorial [4]. https://usegalaxy.eu/u/helena-rasche/h/circos-encode-nature-cover

Workflow Summarisation

Visualisations in the Galaxy framework are usually implemented as interactive JavaScript components, but these plots cannot be created automatically in workflows. Individual plot-

ting tools exist as Galaxy tools, however these are less common and generally less flexible as tool authors must make a trade-off between development time and feature support. We put significant time into the development in order to make an extremely generic tool, enabling researchers to use the Galactic Circos tool in their workflows, based on previous experiences building single-purpose Circos plotting tools (e.g. as in Figure 4). This enables creation of human-readable summaries of large analysis workflows, similar to the non-genomics focused iReport [17]. Galactic Circos was born from precisely this use-case, and therefore aims to enable reducing complex analysis pipeline outputs, such as the workflows required in cancer genomics, allowing bioinformaticians to produce a single image summarising all of their relevant outputs in an easily digestible manner.

Supporting Tools

Circos requires input datasets to adhere to a specific and custom file format. In order to facilitate the conversion of data to this custom Circos format, we have developed several supporting Galaxy tools for conversion. These tools allow users to convert their datasets from a variety of common genomics formats such as (big)Wig files, interval files, and MAF/Stockholm alignments. Furthermore, the existing Galaxy ecosystem provides a wide array of tabular data manipulation tools that can be leveraged to transform any tabular or text files into the format accepted by Circos.

To demonstrate the utility of these supporting tools, we show a real-world example of a plot using common genomics datasets. This example is a recreation of a plot in a published paper demonstrating chromothripsis in the VCaP prostate cancer cell line [12]. The input datasets originate from a variety of sources, including a structural variants files (converted to Circos links track), copy number and B-allele frequency track obtained from Affymetrix SNP array data, and a SNP density track generated from a VCF file. Using a combination of the supporting tools included in the Galactic Circos package and the generic file manipulation tools present in Galaxy, we were able to convert these various datasets to Circos-compatible formats without leaving Galaxy, and reproduced the Circos plot from the publication (Figure 4).

Once data has been reformatted for Circos, it can either be used immediately or be further processed. Circos includes a tool suite for post-processing and downsampling of data which can improve plot clarity and processing speed. We additionally included a number of these post-processing tools into Galaxy, notably the link bundling and binning tools used in Figure 5.

Finally, while Circos is widely used for the visualization of genomic data, and many of the parameter names have a distinctly biological feel to them, the tool does not impose any restrictions on the type of input data, and is capable of displaying non-biological data just as easily [9]. To show that our tool retains this degree of flexibily, we recreated the presidential debate plot included in the Circos tutorials, which in turn was based on a plot which appeared in the New York Times aticle [6]. A plot comparison can be seen in Figure 6.

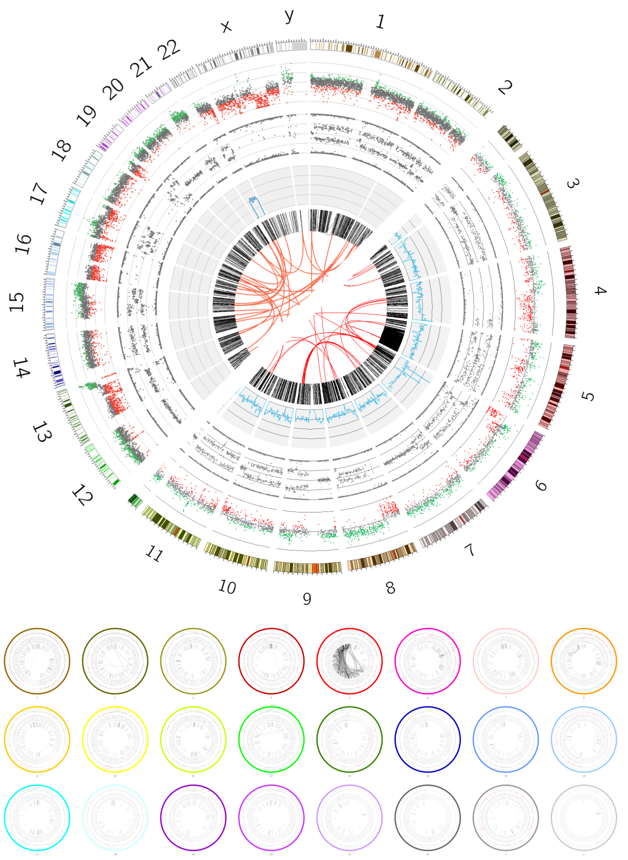

Figure 4. This figure compares output of a custom written Circos plot with hardcoded configuration (top left half), to the output created using the Galactic Circos tool (bottom right half). While the input data originated from a range of standard and nonstandard genomic file formats, conversion to circos-formatted files was possible using the plethora of file manipulation tools already integrated into Galaxy and the set of supporting conversion tools included in the Galactic Circos package. In the second image we produce Circos plots per chromosome, leveraging Galaxy's ability to map a tool execution across a collection of input datasets, in this case each karyotype in a separate input file. The images are reduced and placed together in a montage using further Galaxy tools. https://usegalaxy.eu/u/helena-rasche/h/circos-cancer-genomics--chromothripsis, https://usegalaxy.eu/u/helena-rasche/h/circos-multiplot

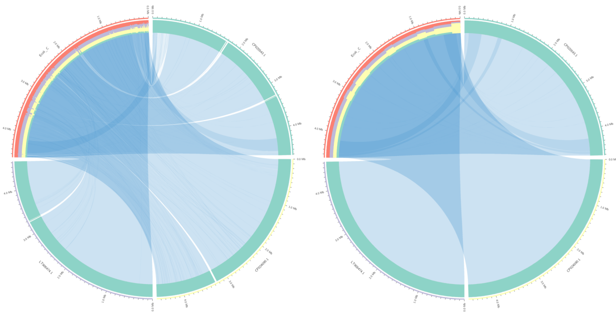

Figure 5. These two plots show the link binning and bundling scripts used with different thresholds. The inner link track was generated directly from a MAF file output by LastZ [20]. This file was processed by Circos' bundling tool in Galaxy in order to decrease the number of links, a process usually done to decrease visual noise and increase efficiency. The outer track demonstrates the link binning script which generates a histogram, in this case from the number of links to that position in the genomic region.

Methods

Implementation

The execution of the tool leverages Galaxy's ability to write templated files directly to disk with configuration from the tool form, and then running Circos directly on these templated configuration files.

Installation of the Circos tool and its dependencies is handled by the Galaxy platform which supports different dependency management frameworks, inlcuding Conda and Containers. All dependencies including circos itself are available from the Bioconda Conda channel [15] and available as a virtualised

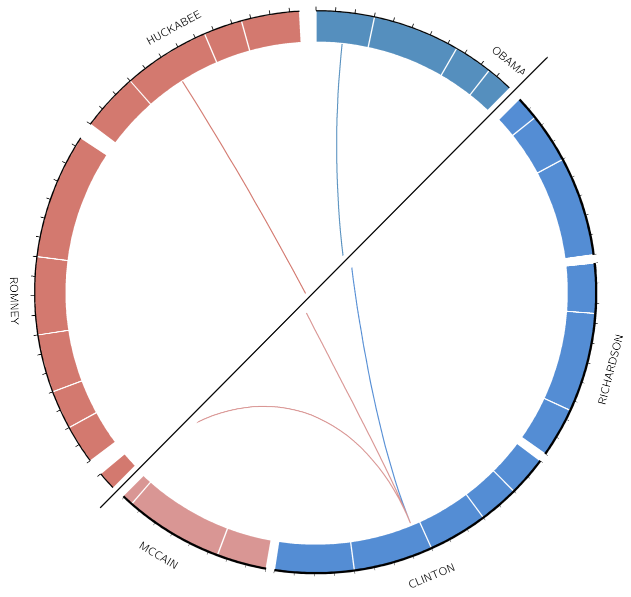

Figure 6. This figure compares the Circos plot from the official tutorial (top left half), to the output created using the Galactic Circos tool (bottom right half). Each link represents a candidate speaking the last name of another candidate. The length of each circle segment is proportional to the total number of words spoken by the candidate during the debates. https://usegalaxy.eu/u/saskia/h/circos-politics-plot

container (rkt, Docker, Singularity). The version of the Galaxy Circos tool being reported on here uses Circos version 0.69.8.

File Format Converters

In order to facilitate the interoperability with upstream tools and workflows, we provide a set of file format converters, in addition to many tools already available in Galaxy, which together provide for convertion of a range of common data format standards (e.g. VCF, MAF/Stockholm, BED/GFF3, BigWig). These tools produce files that are ready to be used as input to the Galaxy Circos tool. Additionally the applicable subset of circos-utils were included into Galaxy for Circos-friendly tools for data reshaping.

Circos Configuration Export

While Galactic Circos aims to offer the full range of Circos functionality, some manual tweaking of the Circos plot configuration files may still be desired. To this end, our tool also outputs the full set of configuration files needed to recreated the plot on the command line, and thus allow easy access to any features not exposed in the Galaxy wrapper.

Training Materials

Our tool greatly simplifies the creation of Circos plots, but the great number of options offered by the Circos tool require good documentation and explanation in order to optimize their utility for end-users. Circos offers a collection of tutorials that are designed to familiarize users with the various features of Circos [3]. In a similar fashion, we have created a set of Galaxy tutorials aimed to educate users in the use of Circos within Galaxy. These tutorials are available from the Galaxy training materials website [13].

Reproducible and Reusable Plots

To enable readers to examine the complete parameters settings used and recreate the example plots given here, Galaxy histories for all the figures shown in this work have been made publicly available from the European Galaxy server (see Availability section).

Future Work

While we have aimed to make our tool as feature-complete as possible, some of Circos' functionality is not currently exposed in the Galaxy tool. We intend to extend our tool to include these features, including but not limited to support for scaling subsections of the plots, and generation of HTML image maps.

Availability of source code and requirements

- Project name: Galactic Circos
- Github repository: https://github.com/galaxyproject/tools-iuc/tree/master/tools/circos
- Tool Shed repository: https://toolshed.g2.bx.psu.edu/view/iuc/circos
- Training Manual: https://training.galaxyproject.org/training-material/topics/visualisation/tutorials/circos/tutorial.html
- Operating system(s): Unix (Platform independent with Docker)
- Other requirements: Galaxy version 18.01 or higher
- License: MIT

The Circos example plots presented in this work are available as Galaxy histories:

- Galaxy history for Figure 2: https://usegalaxy.eu/u/helena-rasche/h/circos-microbe-tutorial
- Galaxy history for Figure 3: https://usegalaxy.eu/u/helena-rasche/h/circos-encode-nature-cover
- Galaxy history for Figure 4a: https://usegalaxy.eu/u/helena-rasche/h/circos-cancer-genomics--chromothripsis
- Galaxy history for Figure 4b: https://usegalaxy.eu/u/helena-rasche/h/circos-multiplot
- Galaxy history for Figure 6: https://usegalaxy.eu/u/saskia/h/circos-politics-plot

Availability of supporting data and materials

The data presented here to illustrate our application was obtained from previous publications, and has been collected and made available from Zenodo [16].

Declarations

List of abbreviations

- VCF: Variant Call Format
- MAF: Multiple Alignment Format

Competing Interests

The authors declare that they have no competing interests.

Funding

This project was made possible with the support of the Albert Ludwig University of Freiburg and German Federal Ministry of Education and Research [031 L0101C de.NBI-epi].

Funding for open access charge: German Federal Ministry of Education and Research.

This project has received funding from the European Union's Horizon 2020 research and innovation programme under grant agreement 825775.

Author's Contributions

HR and SH contributed equally to the tool development, documentation, and writing of the manuscript.

Acknowledgements

The authors would like to thank the Galaxy community for their help in reviewing, testing, and validating the tools presented here.

References

1. Circos Encode Nature cover image Lesson;. http://www.circos.ca/documentation/tutorials/recipes/nature_cover_encode/.
2. Circos Microbial Genome Lesson;. http://www.circos.ca/documentation/tutorials/recipes/microbial_genomes/images.
3. Circos Tutorials;. http://circos.ca/tutorials/lessons/.
4. Circos Tutorials: Recipes - Nature Cover Encode Diagram;. http://www.circos.ca/documentation/tutorials/recipes/nature_cover_encode/images.
5. Galaxy Tool Shed;. https://toolshed.g2.bx.psu.edu/.
6. Naming Names - Interactive Graphic - NYTimes.com;. http://archive.nytimes.com/www.nytimes.com/interactive/2007/12/15/us/politics/DEBATE.html.
7. Nature ENCODE cover (Volume 489 Issue 7414);. https://www.nature.com/nature/volumes/489/issues/7414.
8. Scientific Literature Images Created with Circos;. http://circos.ca/images/published/.
9. Using Circos to Visualize Non-Genomic (General) Data;. http://circos.ca/intro/general_data/.
10. Zotero list of Citations of the Galaxy project;. https://www.zotero.org/groups/1732893/galaxy.
11. Afgan E, Baker D, Batut B, Van Den Beek M, Bouvier D, Čech M, et al. The Galaxy platform for accessible, reproducible and collaborative biomedical analyses: 2018 update. Nucleic acids research 2018;46(W1):W537–W544.
12. Alves IT, Hiltemann S, Hartjes T, van der Spek P, Stubbs A, Trapman J, et al. Gene fusions by chromothripsis of chromosome 5q in the VCaP prostate cancer cell line. Human genetics 2013;132(6):709–713.
13. Batut B, Hiltemann S, Bagnacani A, Baker D, Bhardwaj V, Blank C, et al. Community-Driven Data Analysis Training for Biology. Cell Systems 2018 jun;6(6):752–758.e1. https://doi.org/10.1016/j.cels.2018.05.012.
14. Consortium EP, et al. The ENCODE (ENCyclopedia of DNA elements) project. Science 2004;306(5696):636–640.
15. Grüning B, Dale R, Sjödin A, Chapman BA, Rowe J, Tomkins-Tinch CH, et al. Bioconda: sustainable and comprehensive software distribution for the life sciences. Nature methods 2018;15(7):475.
16. Hiltemann S, GTN Tutorial: Visualization with Circos. Zenodo; 2020. https://zenodo.org/record/3603221.
17. Hiltemann S, Hoogstrate Y, van der Spek P, Jenster G, Stubbs A. iReport: a generalised Galaxy solution for integrated experimental reporting. GigaScience 2014 oct;3(1). https://doi.org/10.1186%2F2047-217x-3-19.
18. Hiltemann S, Mei H, de Hollander M, Palli I, van der Spek P, Jenster G, et al. CGtag: complete genomics toolkit and annotation in a cloud-based Galaxy. GigaScience 2014;3(1):1.
19. Krzywinski M, Schein J, Birol I, Connors J, Gascoyne R, Horsman D, et al. Circos: An information aesthetic for comparative genomics. Genome Research 2009 jun;19(9):1639–1645. https://doi.org/10.1101/gr.092759.109.
20. Rahmani AM, Liljeberg P, Plosila J, Tenhunen H. Lastz: An ultra optimized 3d networks-on-chip architecture. In: 2011 14th Euromicro Conference on Digital System Design IEEE; 2011. p. 173–180.
