# Supplementary material for: Galactic Circos: User-friendly Circos plots within the Galaxy platform
Source: Gigascience. 2020 Jun 12;9(6):giaa065. doi: 10.1093/gigascience/giaa065 (PMC7291503; doi:10.1093/gigascience/giaa065)
Supplement: giaa065_GIGA-D-20-00031_R1 [file giaa065_giga-d-20-00031_r1.pdf]

GigaScience

Galactic Circos: User-friendly Circos Plots within the Galaxy platform

--Manuscript Draft--

**Manuscript Number:**	GIGA-D-20-00031R1
**Full Title:**	Galactic Circos: User-friendly Circos Plots within the Galaxy platform
**Article Type:**	Technical Note
**Funding Information:**	European Union Horizon 2020 (825775) — Ms. Saskia Hiltemann<br>German Federal Ministry of Education and Research (031 L0101C de.NBI-epi) — Ms. Helena Rasche
**Abstract:**	Background: Circos is a popular software package for the circular visualization of complex datasets. Circos is a highly flexible tool. While especially popular in the field of genomic analysis, Circos enables interactive graphing of any analytical data, including alternative scientific domain data and non-scientific data. This high degree of flexibility also comes with a high degree of complexity, which may present an obstacle for researchers not trained in programming or the UNIX command line. The Galaxy platform provides a user-friendly graphical interface incorporating a broad range of "wrapped" command-line tools to facilitate accessibility.<br>Findings: We have developed a Galaxy wrapper for Circos, thus combining the power of Circos with the accessibility and ease-of-use of the Galaxy platform. The combination significantly simplifies the specification and configuration of Circos plots for end-users while retaining the power to produce publication-quality visualizations of complex multidimensional datasets.<br>Conclusions: Galactic Circos enables the creation of publication-ready Circos plots within the Galaxy platform. Users may download the full set of Circos configuration files of their plots for further manual development. This version of Circos is available as an open-source installable application from the Galaxy ToolShed, with its usage clarified in a training manual hosted by the Galaxy Training Network.
**Corresponding Author:**	Helena Rasche<br>Bioinformatics Group, University of Freiburg<br>Freiburg, Baden-Württemberg GERMANY
**Corresponding Author Secondary Information:**	
**Corresponding Author's Institution:**	Bioinformatics Group, University of Freiburg
**Corresponding Author's Secondary Institution:**	
**First Author:**	Helena Rasche
**First Author Secondary Information:**	
**Order of Authors:**	Helena Rasche<br>Saskia Hiltemann
**Order of Authors Secondary Information:**	
**Response to Reviewers:**	# Comments to Editor<br><br>>please register your new software application in the bio.tools and SciCrunch.org databases<br><br>We have registered bio.tools and RRID identifiers and used those in the manuscript. As Galaxy now provides a way to include bio.tools identifiers directly in tool XML, we have done that as well.<br>Reviewer #1<br>Thanks for your very useful and detailed review! We have updated the tool to incorporate your suggestions (except the one or two suggestions we could not (yet)

implement due to security reasons), along with some additional changes we hope will make the tool more user-friendly. We have also updated the training manual to match. The latest version of the tool is now available from the Galaxy ToolShed, and will be available on the European Galaxy server after May 2, for your review (https://usegalaxy.eu). Please feel free to contact us with further suggestions in the future, as both Circos and the Galaxy wrapper continue to evolve.

Below we respond to each of your comments in more detail. To view the code changes to the tool, please see the GitHub pull request here: https://github.com/galaxyproject/tools-iuc/pull/2943

Reviewer #1

1. Karyotype bands

Thanks for these suggestions. We have updated the tool to set 'show_bands = yes" by default, so that when bands are included in the karyotype file, they are now shown by default. Furthermore, Circos provides a number of example karyotype files for a number of model organisms; we now allow users to select one of these presets for their plot. This should make it easier for users to get started with the tool, by eliminating the need to provide a karyotype file themselves.

Given your observation that users familiar with Circos will be accustomed to providing bands in the karyotype file, combined with the addition of an option to select preset karyotype files (which include bands), we have made this the default way to specify bands, and removed the little-used option to provide a separate cytogenetic bands (BED) file. The tool will output the karyotype file to users upon request, to assist in the creation of their own custom karyotype files, by providing an example of the required file format.

2. Add type=highlight 2d track

> This track behaves exactly like a heatmap, except that it does not map the value of the data point onto a color. Instead, it uses the fill_color parameter.

Thanks! We had not previously seen this tab of the documentation.

Highlights were supported as a separate section (as <highlight> block0. We took and merged that into the 2d type (<plot> type=highlight). So now in the wrapper and highlights will be data tracks with type=highlight, and users can use the z-index option to control plotting over or under other datasets as they like.

Does that sufficiently accomplish this? It seems sub-optimal from our perspective (maintenance) and from user experience to have this track type in two places with nearly identical behaviour except z-indexing? Or are there more differences that might necessitate us re-instating the proper highlight track type.

> The reason why this track is needed is because in the wrapper the heatmap track does not allow specifying a specific fill color for a data point (see below).

This should now be supported with the 'advanced' colour entry option.

3. Add z-index for plots and links

Previously this was only possible via rules, but we agree this is suboptimal and have now added a higher-level parameter to control this. We have fixed this in the latest version of the tool.

4. Add custom fields for plot and links

Unfortunately, as much as we would like to, we cannot currently address some parts of this point, due to the security risks involved in allowing user-provided free-text input in Galaxy.

Powered by Editorial Manager® and ProduXion Manager® from Aries Systems Corporation

We believe that if we provided a free-text field, we would not be able to sufficiently, safely sanitise this, preventing all malicious inputs. This is the reason why we have gone to significant lengths to make it possible to just use dropdowns and sliders to configure Circos, lest the user attempt supplying perl code which might be executed on the server.

If we would include such free-text inputs, the usegalaxy.* servers (and likely many others) would not be able to include the Circos tool due to the potential security implications. In the future, Galaxy is working towards more secure and isolated computation. Some servers have started implementing this, and when this has become the norm, we can update the Circos tool to allow such advanced options.

> There are a variety of settings for plots. Some very useful ones are
>
> minsize = MINSIZE

We have added this option since it sounds quite useful!

> Another is color_mapping for heatmaps

We have added this as well.

5. Fill color (especially for heatmaps)

We have implemented it as a text field. For the above-mentioned security reasons, this input is limited to strings matching [a-z0-9,-]+

6. Rules

> a. All actions (links/2d plots) should support the z-index (or z-depth as the wrapper calls it).

Done! And we have updated the text to z-index.

> b. There is a bug in how a rule interprets "change fill color based on value" when "0"

Fixed

> The wording "based on numerical position" is not intuitive and should be "based on value". Here, "position" can be easily confused with genomic position.

How about “Based on position on chromosome” which is the intention, and “based on position value” for the other? We have made this change, but if there is a better label, we can substitute that.

> The wording "expected minimum value of dataset" is not intuitive. It should be … "for points below value" … "for points above value"

We’ve changed these to just “minimum / maximum value” with a better explanation in the brewer scale selector:
Fill will be changed based on a mapping of dataset values to colours, but to do this the mapping needs to be defined. Minimum and maximum values are defined below, and will map to the minimum and maximum values of this brewer scale.

> The reason why this is very helpful is that you can combine several parameters in a data point
>
> hs1 10 20 0.5 myvar=0.75,myvar2=1.5,myvar3=2.5,…

Generating files like this is not trivial in Galaxy, so we had not encountered the need to support such a feature yet, most people just generate N data files using Galaxy collections and other features.

We have added support for this in case someone needs it.

Powered by Editorial Manager® and ProduXion Manager® from Aries Systems Corporation

7. Axes & Backgrounds

We recently removed it because some users found it confusing. We have re-added it and reworded it significantly, maybe it'll be clearer now.

8. Loading additional color profiles

This again would be untrusted user input, and would require significant sanitise rules to permit most valid human inputs. Given the additional comment of care needing to be taken to not redefine existing colours, implementing this would require significant work to ensure a good user experience. I am not sure it is possible to do this, and unfortunately we will have to relegate such a feature to being out of scope for the intention of the wrapper.

If we could identify some commonly used colour profiles we could easily build those into the tool?

9. Custom fields in each block

> Each block in the configuration file should have an option for a set of custom field definitions. This nicely gets around any functionality that the wrapper doesn't support.

see #4

> chromosomes_order

This is supported in the chromosome limit setting under Ideogram options. We decided to merge the limiting and ordering as a simplification for users.

This unfortunately comes again at the expense of limiting user terseness (if they want all the chromosomes with an order they just have to write it all), but at least in terms of Galaxy it should make the tool slightly more fool proof for untrained users.

We've updated the documentation to mention this additional ordering that is happening, so hopefully it is more clear.

10. Ideogram colors

> The "ideogram color scheme" currently isn't intuitive

This behaviour is specific to a couple of track types, and yes, misleading. We have reworked it, now the option is only visible for the tracks where it can be used (fasta to karyotype, lengths file to karyotype). And it won't be shown for a standard karyotype input where the colours won't be overridden. Additionally when the user uses a fasta or length file as input, we now output the karyotype file so they can re-use this karyotype file directly rather than having that be re-calculated every time. Hopefully this will get non-Circos familiar users more experience with Circos' karyotype files.

> The wrapper behaves strangely when not auto-detecting a file as BED. If I upload a .txt file (e.g. space-delimited) and not explicitly convert it to BED then it doesn't show up in the file dropdown list. When using the folder icon next to the dropdown list the file is prefixed with "(unavailable)".

Ah there are two folder icons, on the left is the "Collection input" (not relevant here), on the right is the new dataset selector. In this case, it cannot auto-convert the datatypes I guess, so, it refuses to accept this input.

Important

> I don't think it's important to insist on BED (which is a loose format anyway).
> When viewing the data file in galaxy, the entire line shows up in the first column.

Powered by Editorial Manager® and ProduXion Manager® from Aries Systems Corporation

For us, one of Galaxy’s main selling points is that users cannot provide really wrong inputs to a tool, e.g. you cannot select a PNG file where a table input is required. Likewise, you cannot provide a generic table, when we know that the tool needs an Interval format file (bed3 + any number of columns after).

By enforcing the permitted input file types we help ensure our users cannot provide wrong inputs and remove a class of potential support issues we would have to solve on a regular basis.

Additionally, given that numerous other Galaxy tools exist to process tabular data (cutting rows and columns, computing expressions on columns, etc.), and they all have a hard requirement on tab-separated data, we fear we would be hurting the user’s potential Galaxy experience, if we permitted .txt files, and they could only send them to Circos, and were unable to use them in many tools or pipelines that might exist on those servers.

Space separated data can be converted on upload with the built in option of converting spaces to tabs (under gear icon) if this helps?

TODO

> Add connector track (2d track type).

Done!

Additionally we have implemented the “Zoom” feature since we stumbled across it in the documentation and it looked very useful.

Reviewer #2

Thanks a lot for your valuable comments and insights. We have updated the manuscript according to your comments. More detailed point-by-point responses are given below.

> Choose to use either "visualisation" or "visualization"

We have replaced all british-isms with american english for consistency.

> Consider reformatting manuscript title to aid with searches (yes, this is more formal!): "Galactic Circos: User-friendly Circos Plots within the Galaxy platform".

Done! Thanks for the tip.

> Consider the use of upper case "X" and "Y" chromosome nomenclature in Figure 4 (and in the underlying input data at Zenoto, if possible):

That side of the image was the original image appearing in a previous publication which we were reproducing with our tool, so we cannot change that part of the image unfortunately. However, we do agree with you that uppercase should be the default. As part of this revision, we added a set of preconfigured karyotype files for model organisms to the tool, so that users who use one of these model organisms do not have to supply a karyotype file. In these presets, the X and Y chromosomes have uppercase labels, so this should be the default behaviour of the tool now, unless users manually supply a karyotype file with lowercase chromosome labels.

> Consider alternating formal and informal variations of verbiage: The term "tweak" may be a bit overused

Thanks, we have reduced the use of the word tweaking, and hope it is more aligned with Circos nomenclature now.

> Consider consistent naming when referencing the Galaxy ToolShed:

Powered by Editorial Manager® and ProduXion Manager® from Aries Systems Corporation

	Done!<br><br>> Consider adding in links and resources for all items listed under "Availability of source code and requirements".<br><br>Great idea, we have added your suggested links (and a few additional ones).<br><br># Reviewer #3<br><br>Thank you for your review, we have addressed the concerns you raised. We have added a section describing the lessons learned and listing more explicitly the limitations of the tool. We have also updated the sharing settings for the inaccessible history link. Regarding the figures, we have high-quality source images for all figures, and will confer with the editors about the best placement of these, since the current configuration was dictated by the LaTeX template, but may be altered for the final publication.<br><br>> To the best of my knowledge, this is one of the more comprehensive efforts to integrate a visualization package into Galaxy or similar GUI workflow platform. Given the unique aspect of this work, it would useful for the manuscript to have a section discussing lessons learned as well as limitations of the tool.<br><br>We have added a section describing numerous lessons learnt in the paper, attempting to filter out those which would be useful for an average reader. There are numerous additional issues or pain points which could be expounded upon but would be too technical in this context. We can open discussions with the Galaxy team regarding how those might be fixed.
**Additional Information:**	
**Question**	**Response**
Are you submitting this manuscript to a special series or article collection?	No
**Experimental design and statistics**<br><br>Full details of the experimental design and statistical methods used should be given in the Methods section, as detailed in our Minimum Standards Reporting Checklist. Information essential to interpreting the data presented should be made available in the figure legends.<br><br>Have you included all the information requested in your manuscript?	No
If not, please give reasons for any omissions below.<br><br>as follow-up to "**Experimental design and statistics**	No experiment was conducted, we simply report the production of a new piece of software? But if there is more information we can include we would be happy to

Full details of the experimental design and statistical methods used should be given in the Methods section, as detailed in our Minimum Standards Reporting Checklist. Information essential to interpreting the data presented should be made available in the figure legends.<br><br>Have you included all the information requested in your manuscript?<br><br>"	
**Resources**<br><br>A description of all resources used, including antibodies, cell lines, animals and software tools, with enough information to allow them to be uniquely identified, should be included in the Methods section. Authors are strongly encouraged to cite Research Resource Identifiers (RRIDs) for antibodies, model organisms and tools, where possible.<br><br>Have you included the information requested as detailed in our Minimum Standards Reporting Checklist?	Yes
**Availability of data and materials**<br><br>All datasets and code on which the conclusions of the paper rely must be either included in your submission or deposited in publicly available repositories (where available and ethically appropriate), referencing such data using a unique identifier in the references and in the "Availability of Data and Materials" section of your manuscript.<br><br>Have you have met the above requirement as detailed in our Minimum Standards Reporting Checklist?	Yes

Powered by Editorial Manager® and ProduXion Manager® from Aries Systems Corporation

Click here to access/download;LaTeX - Figure (eps, ps, etc.);plot-microbe-both.png

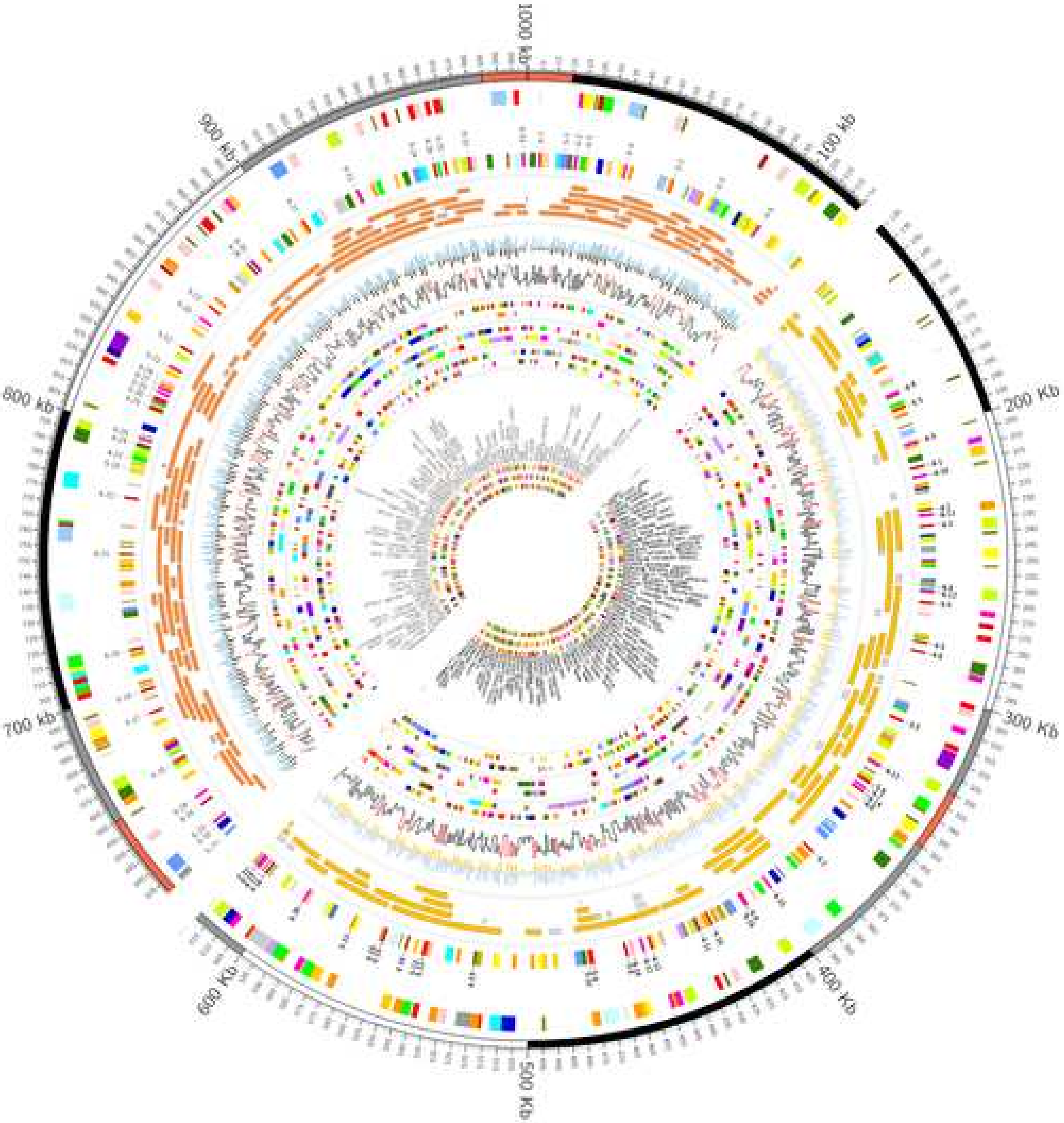

Click here to access/download;LaTeX - Figure (eps, ps, etc.);oup.eps

OXFORD

Click here to access/download;LaTeX - Figure (eps, ps, etc.);gigascience-logo.eps

Manuscript Click here to access/download;Manuscript;main.tex

OXFORD (GIGA)^n SCIENCE

GigaScience, 2017, 1–6

doi: xx.xxxx/xxxx
Manuscript in Preparation
Technical Note

TECHNICAL NOTE

Galactic Circos: User-friendly Circos Plots within the Galaxy platform

Helena Rasche[1,*,†] and Saskia Hiltemann[2,*,†]

[1]Bioinformatics Group, Department of Computer Science, University of Freiburg, 79110 Freiburg im Breisgau, Germany and [2]Erasmus Medical Center, Clinical Bioinformatics group, Department of Pathology, Wytemaweg 80, 3015 CN, Rotterdam, The Netherlands

[*]hxr@informatik.uni-freiburg.de; saskiahiltemann@gmail.com
[†]Contributed equally.

Abstract

Background: Circos is a popular software package for the circular visualization of complex datasets. Circos is a highly flexible tool. While especially popular in the field of genomic analysis, Circos enables interactive graphing of any analytical data, including alternative scientific domain data and non-scientific data. This high degree of flexibility also comes with a high degree of complexity, which may present an obstacle for researchers not trained in programming or the UNIX command line. The Galaxy platform provides a user-friendly graphical interface incorporating a broad range of "wrapped" command-line tools to facilitate accessibility.
Findings: We have developed a Galaxy wrapper for Circos, thus combining the power of Circos with the accessibility and ease-of-use of the Galaxy platform. The combination significantly simplifies the specification and configuration of Circos plots for end-users while retaining the power to produce publication-quality visualizations of complex multidimensional datasets.
Conclusions: Galactic Circos enables the creation of publication-ready Circos plots within the Galaxy platform. Users may download the full set of Circos configuration files of their plots for further manual development. This version of Circos is available as an open-source installable application from the Galaxy ToolShed, with its usage clarified in a training manual hosted by the Galaxy Training Network.

Key words: Genomics; Visualization; Galaxy; Circos; UI/UX

Findings

Background

The Circos visualization tool [19] is widely used in the biological scientific community, and is especially popular for use in scientific publications. Circos has over 4000 citations, and its plots have appeared on the cover of several leading scientific journals [8]. Its popularity is due in a large part to its great flexibility; Circos offers a wide range of visualization options, and all aspects of a Circos plot may be customized to the user's needs. While originally created for the visualization of genomic data, Circos makes no *a priori* assumptions about the format and domain of the input data; this is illustrated by the fact that it has been used for a wide range of applications, ranging from genomics research to visualizations of car sales, urban planning, and even presidential debates [9].

With Circos's great flexibility also comes a high degree of complexity, and a significant learning curve, and as a result its use is often limited to expert users who are experienced with programming and the UNIX command line.

The Galaxy platform [11] aims to provide a user-friendly interface to commandline tools, and empower domain experts to run powerful analysis and visualization tools without the need

Compiled on: April 30, 2020.
Draft manuscript prepared by the author.

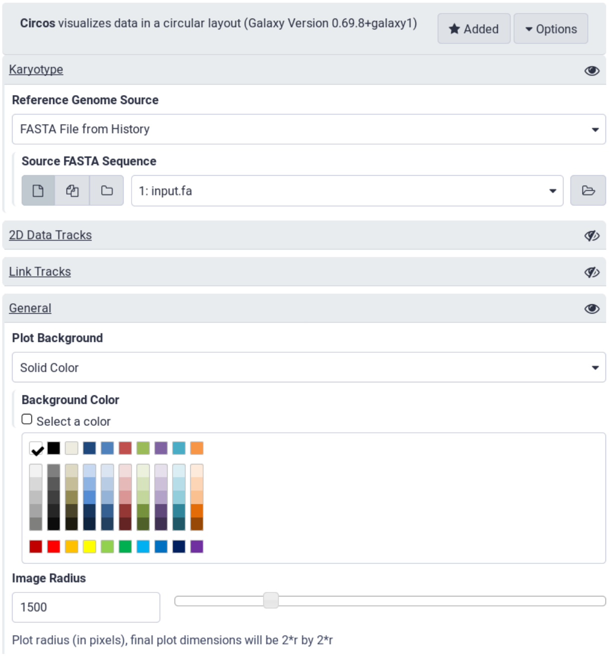

Figure 1. The Galaxy tool interface to Circos. Each collapsed section hides a wealth of configuration options available to users. The web based interface is significantly more accessible than the command line version.

for any programming experience. Galaxy offers a wide range of tools for a variety of applications domains, and is widely used in the biological scientific community (8900+ citations, 7500+ tools [10, 5]). Galaxy also automates the installation of tools and all their dependencies, removing another hurdle for its use by research scientists.

Our tool combines the power of Circos with the user-friendliness of the Galaxy interface to greatly increase the accessibility of the tool and simplify the creation of publication-ready plots for scientific data.

Previously, custom Circos Galaxy plotter tools have been written [18]; however, these tools are not generic, but are tailored specifically to the use case at hand. This means that a new Galaxy tool has to be created whenever a new plot type is needed. Galactic Circos aims to be a generic tool capable of creating any Circos plot regardless of data domain.

Results

The Galactic Circos tool changes the way users must specify the configuration of a Circos plot. Instead of writing a number of configuration files, users now only need to select the various plot options from a web interface, and datasets from their analysis history (Figure 1). Because Circos plot specifications can be quite complex, the tool interface is subdivided into several collapsible sections, each corresponding to a different Circos configuration option in order to increase the usability of the tool. Parameters are pre-configured with sensible default values so that basic plots can be generated with minimal configuration.

We demonstrate the utility of the Galactic Circos tool by recreating one of the more advanced examples from the Circos online tutorials, the microbial genome lesson [2] (Figure 2). This displays multiple tracks of different types (text, histogram, tiles), has a customized ideogram, and uses rules for coloring data points dependent on their value.

In a second example (Figure 3), we replicate within Galaxy the cover image of the Nature issue [7] dedicated to the ENCODE project [14]. This cover featured a Circos plot and is also available as part of the official Circos tutorials [1].

Figure 2. Here we reproduce one of the more complex tutorials from the Circos documentation. The top-left half of the image is produced by the configuration provided by the Circos tutorial, while the bottom-right half is produced completely in Galaxy. While some options used in the original tutorial cannot be directly used (e.g. unrestricted Perl code), they can be recreated equivalently in the tool interface. Some options in the tool interface are likewise restricted, Galactic Circos offers a color picker with a limited palette, which explains the differences in color. However, our tool offers the ability to download the full Circos configuration folder, allowing advanced users to configure the color (or other) parameters manually and rebuild the image locally. https://usegalaxy.eu/u/helena-rasche/h/circos-microbe-tutorial

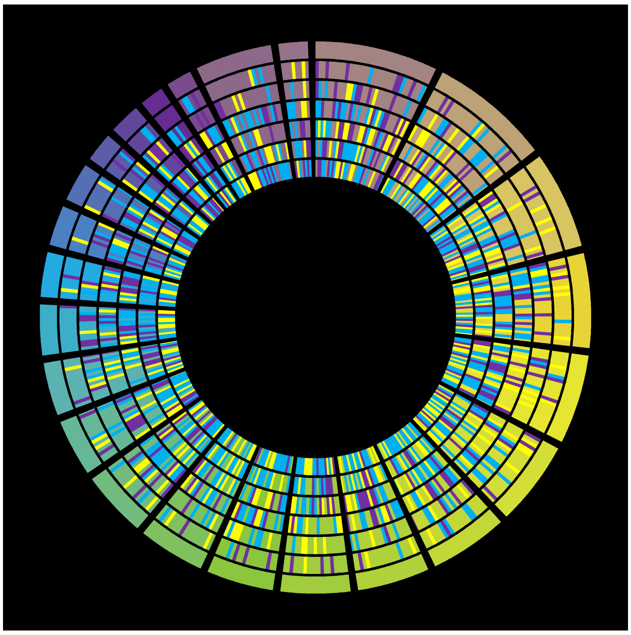

Figure 3. Nature's cover [7] for the ENCODE project in September 2012, reproduced by Galactic Circos. The image is not a split image due to copyright restrictions on the original cover image. Comparison can be made against the Circos tutorial [4]. https://usegalaxy.eu/u/helena-rasche/h/circos-encode-nature-cover

These two examples showcase a variety of different track types (histograms, scatterplot, highlights, tiles, text) and configurations (ticks, rules, ideogram customizations) to illustrate the feature-completeness of Galactic Circos.

Workflow Summarization

Visualizations in the Galaxy framework are usually implemented as interactive JavaScript components, but these plots cannot be created automatically in workflows. Individual plotting tools exist as Galaxy tools, however these are less common and generally less flexible as tool authors must make a trade off between development time and feature support. We put significant time into the development in order to make an extremely generic tool, enabling researchers to use the Galactic Circos tool in their workflows, based on previous experiences building single-purpose Circos plotting tools (e.g. as in Figure 4). This enables creation of human-readable summaries of large analysis workflows, similar to the non-genomics focused iReport [17]. Galactic Circos was born from precisely this use-case, and therefore aims to enable reducing complex analysis pipeline outputs, such as the workflows required in cancer genomics, allowing bioinformaticians to produce a single image summarizing all of their relevant outputs in an easily digestible manner.

Supporting Tools

Circos requires input datasets to adhere to a specific and custom file format. In order to facilitate the conversion of data to this custom Circos format, we have developed several supporting Galaxy tools for conversion. These tools allow users to convert their datasets from a variety of common genomics formats such as (big)Wig files, interval files, and MAF/Stockholm alignments. Furthermore, the existing Galaxy ecosystem provides a wide array of tabular data manipulation tools that can be leveraged to transform any tabular or text files into the format accepted by Circos.

To demonstrate the utility of these supporting tools, we show a real-world example of a plot using common genomics datasets. This example is a recreation of a plot in a published paper demonstrating chromothripsis in the VCaP prostate cancer cell line [12]. The input datasets originate from a variety of sources, including a structural variants files (converted to Circos links track), copy number and B-allele frequency track obtained from Affymetrix SNP array data, and a SNP density track generated from a VCF file. Using a combination of the supporting tools included in the Galactic Circos package and the generic file manipulation tools present in Galaxy, we were able to convert these various datasets to Circos-compatible formats without leaving Galaxy, and reproduced the Circos plot from the publication (Figure 4).

Once data has been reformatted for Circos, it can either be used immediately or be further processed. Circos includes a tool suite for post-processing and down-sampling of data which can improve plot clarity and processing speed. We additionally included a number of these post-processing tools into Galaxy, notably the link bundling and binning tools used in Figure 5.

Finally, while Circos is widely used for the visualization of genomic data, and many of the parameter names have a distinctly biological feel to them, the tool does not impose any restrictions on the type of input data, and is capable of displaying non-biological data just as easily [9]. To show that our tool retains this degree of flexibly, we recreated the presidential debate plot included in the Circos tutorials, which in turn was based on a plot which appeared in the New York Times article [6]. A plot comparison can be seen in Figure 6.

Lessons learned and limitations

Given the great flexibility and configurability of the Circos tool, our Galaxy wrapper is, to our knowledge, one of the most complex Galaxy tools. Development of this wrapper took significant time and resources, and in places took us to the edges of what is possible in Galaxy. In this section we describe some of the lessons learned and tips for wrapping tools of this complexity.

Security

We mentioned previously that this wrapper exposes $\approx$95% of what is possible with Circos. We intentionally excluded the last $\approx$5% as we could not safely implement it. These features would require allowing free-text user input files, which pose a potential security risk. We felt we could not, within a reasonable period of development time, implement fool-proof sanitization of all possible user inputs. Instead, we provide an option for the tool to output the full set of configuration files required to recreate the plot, which the user can use as a starting point for manual adaptation locally. There are ongoing efforts within the Galaxy community to do computations with increasingly untrusted user input, and we hope that the Galaxy community will push this even further in the future and make it the default, rather than requiring special configuration and knowledge from system administrators. This would enable us to add a free-text field within the Circos tool, and users could provide custom configuration freely and without risk to the adminis-

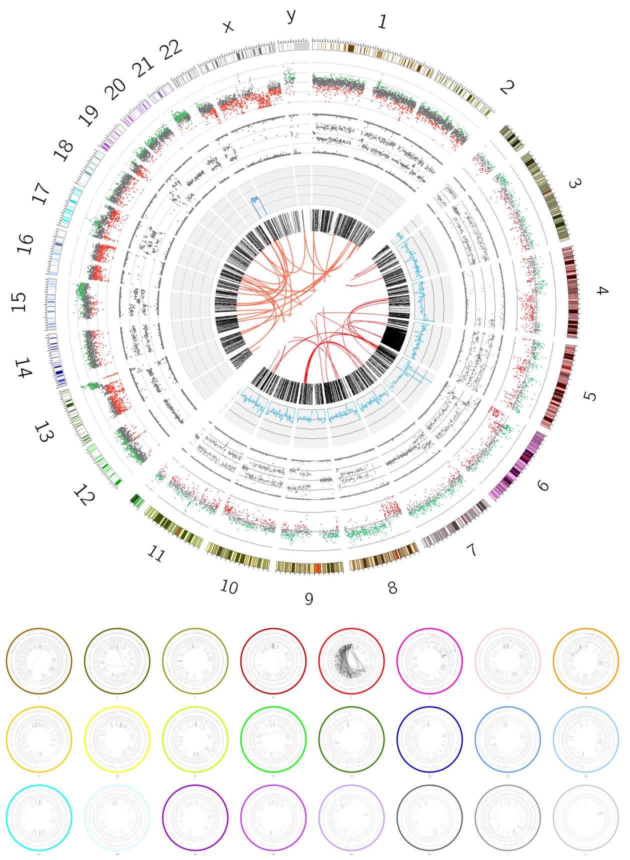

Figure 4. This figure compares output of a custom written Circos plot with hard coded configuration (top left half), to the output created using the Galactic Circos tool (bottom right half). While the input data originated from a range of standard and nonstandard genomic file formats, conversion to Circos-formatted files was possible using the plethora of file manipulation tools already integrated into Galaxy and the set of supporting conversion tools included in the Galactic Circos package. In the second image we produce Circos plots per chromosome, leveraging Galaxy's ability to map a tool execution across a collection of input datasets, in this case each karyotype in a separate input file. The images are reduced and placed together in a montage using further Galaxy tools. https://usegalaxy.eu/u/helena-rasche/h/circos-cancer-genomics--chromothripsis, https://usegalaxy.eu/u/helena-rasche/h/circos-multiplot

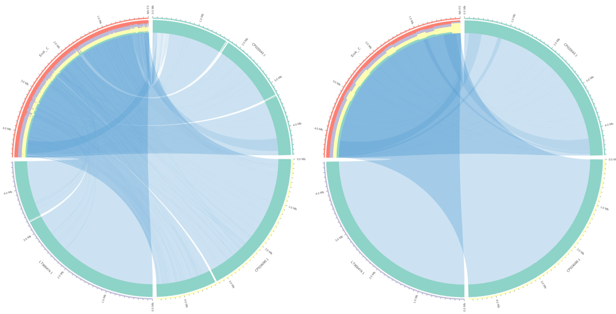

Figure 5. These two plots show the link binning and bundling scripts used with different thresholds. The inner link track was generated directly from a MAF file output by LastZ [20]. This file was processed by Circos' bundling tool in Galaxy in order to decrease the number of links, a process usually done to decrease visual noise and increase efficiency. The outer track demonstrates the link binning script which generates a histogram, in this case from the number of links to that position in the genomic region.

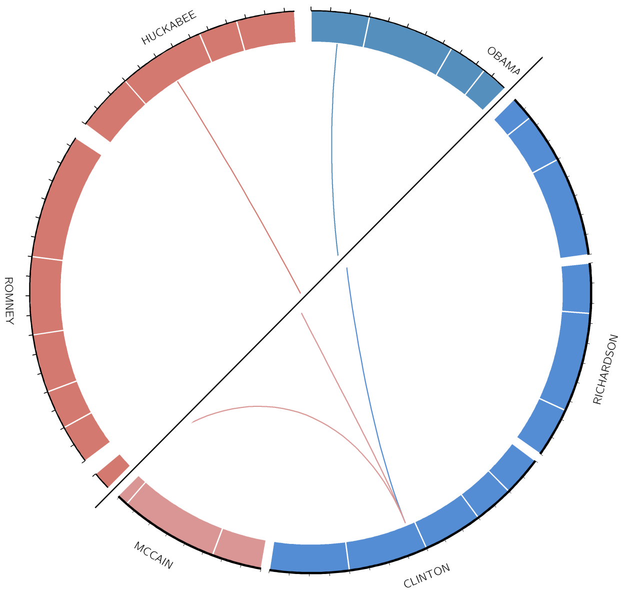

Figure 6. This figure compares the Circos plot from the official tutorial (top left half), to the output created using the Galactic Circos tool (bottom right half). Each link represents a candidate speaking the last name of another candidate. The length of each circle segment is proportional to the total number of words spoken by the candidate during the debates. https://usegalaxy.eu/u/saskia/h/circos-politics-plot

trator.

Visualization vs. Tool

We made the initial choice to build Galactic Circos as a tool, not a visualisation given the long compilation times of plots and our desire to build a workflow compatible tool as this was not possible in Galaxy at that time. In the future, we might want to explore the possibilities of a more dynamic visual interface, using a vizualization plugin in Galaxy. We would have complete freedom to build in more interactivity and custom components (e.g. for Brewer scale selection) as needed.

Macros

Macros proved incredibly helpful in wrangling the complexity of this tool by allowing us to define reusable components and avoid code duplication. Galaxy wrappers allow for the definition of *macros*; these are bits of code defined in a file outside the main wrapper, and can be re-used at multiple points in the tool. Unfortunately, with the extent to which this tool relies on macros, it also makes the tool more complex from a development point-of-view, with the code spread out over a large numbe of files. However, the benefits here outweigh the drawbacks. Furthermore, we came across a number of features in the tool where code was near-identical, but not 100% the same, where macros were not possible.

Collapsible Sections

The section feature in Galaxy permits grouping related options together in the user interface. This avoids overwhelming the user with the enormous array of available parameters, but rather groups these logically and only shows those subsets requested by the user. Unfortunately, these sections re-collapse themselves during tool re-run, and are not marked when their children contain modifications from the defaults. If either of these was changed, users could more easily recall what they did in the previous tool run, as all edited sections would be expanded, or marked by default.

Color

The built in color selector provides a small palette of colors. While it is a good thing to prevent users from making plots with hard to see or unpleasant colors, it also significantly limits more advanced users. The addition of an RGB/HSV color picker would be welcome for Circos users. Likewise we used a select box for Brewer palette which feels sub-optimal compared to a component which includes a preview of that palette and would be much more user-friendly.

As we have mentioned before, while Galactic Circos supports the major features present in Circos, some of our decisions were made in favor of usability,

Methods

Implementation

The execution of the tool leverages Galaxy's ability to write templated files directly to disk with configuration from the tool form, and then running Circos directly on these templated configuration files.

Installation of the Circos tool and its dependencies is handled by the Galaxy platform which supports different dependency management frameworks, including Conda and Containers. All dependencies including Circos itself are available from the Bioconda Conda channel [15] and available as a virtualized container (rkt, Docker, Singularity). The version of the Galaxy Circos tool being reported on here uses Circos version 0.69.8.

File Format Converters

In order to facilitate the interoperability with upstream tools and workflows, we provide a set of file format converters, in addition to many tools already available in Galaxy, which together provide for conversion of a range of common data format standards (e.g. VCF, MAF/Stockholm, BED/GFF3, BigWig). These tools produce files that are ready to be used as input to the Galaxy Circos tool. Additionally the applicable subset of circos-utils were included into Galaxy for Circos-friendly tools for data reshaping.

Circos Configuration Export

While Galactic Circos aims to offer the full range of Circos functionality, some manual customization of the Circos plot configuration files may still be desired. To this end, our tool also outputs the full set of configuration files needed to recreated the plot on the command line, and thus allow easy access to any features not exposed in the Galaxy wrapper.

Training Materials

Our tool greatly simplifies the creation of Circos plots, but the great number of options offered by the Circos tool require good documentation and explanation in order to optimize their utility for end-users. Circos offers a collection of tutorials that are designed to familiarize users with the various features of Circos [3]. In a similar fashion, we have created a set of Galaxy tutorials aimed to educate users in the use of Circos within Galaxy. These tutorials are available from the Galaxy training materials website [13].

Reproducible and Reusable Plots

To enable readers to examine the complete parameters settings used and recreate the example plots given here, Galaxy histories for all the figures shown in this work have been made publicly available from the European Galaxy server (see Availability section).

Future Work

While we have aimed to make our tool as feature-complete as possible, some of Circos' functionality is not currently exposed in the Galaxy tool. We intend to extend our tool to include these features, including but not limited to support for scaling subsections of the plots, and generation of HTML image maps.

Availability of source code and requirements

- Project name: Galactic Circos
- bio.tools ID: galactic_circos
- RRID:SCR_018207
- Github repository: https://github.com/galaxyproject/tools-iuc/tree/master/tools/circos
- ToolShed repository: https://toolshed.g2.bx.psu.edu/view/iuc/circos
- Training Manual: https://training.galaxyproject.org/training-material/topics/visualisation/tutorials/circos/tutorial.html
- Operating system(s): Unix (Platform independent with Docker)
- Other requirements: Galaxy version 18.01 or higher
- License: MIT

The Circos example plots presented in this work are available as Galaxy histories:

- Galaxy history for Figure 2: https://usegalaxy.eu/u/helena-rasche/h/circos-microbe-tutorial
- Galaxy history for Figure 3: https://usegalaxy.eu/u/helena-rasche/h/circos-encode-nature-cover
- Galaxy history for Figure 4a: https://usegalaxy.eu/u/helena-rasche/h/circos-cancer-genomics--chromothripsis
- Galaxy history for Figure 4b: https://usegalaxy.eu/u/helena-rasche/h/circos-multiplot
- Galaxy history for Figure 6: https://usegalaxy.eu/u/saskia/h/circos-politics-plot

Galaxy Resources

- Galaxy Home Page: https://galaxyproject.org/
- Galaxy Tutorials: https://training.galaxyproject.org
- How to install Galaxy: https://getgalaxy.org
- How to install tools: https://galaxyproject.org/admin/tools/add-tool-from-toolshed-tutorial/
- Full Administrative resources: https://docs.galaxyproject.org/
- Galaxy Help Forum: https://help.galaxyproject.org/
- Connect with the Galaxy Community on Gitter Chat: https://gitter.im/galaxyproject/Lobby/
- Public Galaxy servers that include Circos: usegalaxy.eu, usegalaxy.org, usegalaxy.org.au (see Galactic Circos tutorial for full up-to-date list)

Availability of supporting data and materials

The data presented here to illustrate our application was obtained from previous publications, and has been collected and made available from Zenodo [16].

Declarations

List of abbreviations

- VCF: Variant Call Format
- MAF: Multiple Alignment Format

Competing Interests

The authors declare that they have no competing interests.

Funding

This project was made possible with the support of the Albert Ludwig University of Freiburg and German Federal Ministry of Education and Research [031 L0101C de.NBI-epi].

Funding for open access charge: German Federal Ministry of Education and Research.

This project has received funding from the European Union's Horizon 2020 research and innovation program under grant agreement 825775.

Author's Contributions

HR and SH contributed equally to the tool development, documentation, and writing of the manuscript.

Acknowledgments

The authors would like to thank the Galaxy community for their help in reviewing, testing, and validating the tools presented here.

References

1. Circos Encode Nature cover image Lesson;. http://www.circos.ca/documentation/tutorials/recipes/nature_cover_encode/.
2. Circos Microbial Genome Lesson;. http://www.circos.ca/documentation/tutorials/recipes/microbial_genomes/images.
3. Circos Tutorials;. http://circos.ca/tutorials/lessons/.
4. Circos Tutorials: Recipes - Nature Cover Encode Diagram;. http://www.circos.ca/documentation/tutorials/recipes/nature_cover_encode/images.
5. Galaxy Tool Shed;. https://toolshed.g2.bx.psu.edu/.
6. Naming Names - Interactive Graphic - NYTimes.com;. http://archive.nytimes.com/www.nytimes.com/interactive/2007/12/15/u
7. Nature ENCODE cover (Volume 489 Issue 7414);. https://www.nature.com/nature/volumes/489/issues/7414.
8. Scientific Literature Images Created with Circos;. http://circos.ca/images/published/.
9. Using Circos to Visualize Non-Genomic (General) Data;. http://circos.ca/intro/general_data/.
10. Zotero list of Citations of the Galaxy project;. https://www.zotero.org/groups/1732893/galaxy.
11. Afgan E, Baker D, Batut B, Van Den Beek M, Bouvier D, Čech M, et al. The Galaxy platform for accessible, repro-

ducible and collaborative biomedical analyses: 2018 update. Nucleic acids research 2018;46(W1):W537–W544.
12. Alves IT, Hiltemann S, Hartjes T, van der Spek P, Stubbs A, Trapman J, et al. Gene fusions by chromothripsis of chromosome 5q in the VCaP prostate cancer cell line. Human genetics 2013;132(6):709–713.
13. Batut B, Hiltemann S, Bagnacani A, Baker D, Bhardwaj V, Blank C, et al. Community-Driven Data Analysis Training for Biology. Cell Systems 2018 jun;6(6):752–758.e1. https://doi.org/10.1016/j.cels.2018.05.012.
14. Consortium EP, et al. The ENCODE (ENCyclopedia of DNA elements) project. Science 2004;306(5696):636–640.
15. Grüning B, Dale R, Sjödin A, Chapman BA, Rowe J, Tomkins-Tinch CH, et al. Bioconda: sustainable and comprehensive software distribution for the life sciences. Nature methods 2018;15(7):475.
16. Hiltemann S, GTN Tutorial: Visualization with Circos. Zenodo; 2020. https://zenodo.org/record/3603221.
17. Hiltemann S, Hoogstrate Y, van der Spek P, Jenster G, Stubbs A. iReport: a generalised Galaxy solution for integrated experimental reporting. GigaScience 2014 oct;3(1). https://doi.org/10.1186%2F2047-217x-3-19.
18. Hiltemann S, Mei H, de Hollander M, Palli I, van der Spek P, Jenster G, et al. CGtag: complete genomics toolkit and annotation in a cloud-based Galaxy. GigaScience 2014;3(1):1.
19. Krzywinski M, Schein J, Birol I, Connors J, Gascoyne R, Horsman D, et al. Circos: An information aesthetic for comparative genomics. Genome Research 2009 jun;19(9):1639–1645. https://doi.org/10.1101/gr.092759.109.
20. Rahmani AM, Liljeberg P, Plosila J, Tenhunen H. Lastz: An ultra optimized 3d networks-on-chip architecture. In: 2011 14th Euromicro Conference on Digital System Design IEEE; 2011. p. 173–180.
