# Supplementary material for: Galactic Circos: User-friendly Circos plots within the Galaxy platform
Source: Gigascience. 2020 Jun 12;9(6):giaa065. doi: 10.1093/gigascience/giaa065 (PMC7291503; doi:10.1093/gigascience/giaa065)
Supplement: giaa065_Response_to_Reviewer_Comments_Original_Submission [file giaa065_response_to_reviewer_comments_original_submission.pdf]

Author's Response To Reviewer Comments

Close

Comments to Editor

>please register your new software application in the bio.tools and SciCrunch.org databases

We have registered bio.tools and RRID identifiers and used those in the manuscript. As Galaxy now provides a way to include bio.tools identifiers directly in tool XML, we have done that as well.
Reviewer #1
Thanks for your very useful and detailed review! We have updated the tool to incorporate your suggestions (except the one or two suggestions we could not (yet) implement due to security reasons), along with some additional changes we hope will make the tool more user-friendly. We have also updated the training manual to match. The latest version of the tool is now available from the Galaxy ToolShed, and will be available on the European Galaxy server after May 2, for your review (https://usegalaxy.eu). Please feel free to contact us with further suggestions in the future, as both Circos and the Galaxy wrapper continue to evolve.

Below we respond to each of your comments in more detail. To view the code changes to the tool, please see the GitHub pull request here: https://github.com/galaxyproject/tools-iuc/pull/2943

Reviewer #1

1. Karyotype bands

Thanks for these suggestions. We have updated the tool to set 'show_bands = yes" by default, so that when bands are included in the karyotype file, they are now shown by default. Furthermore, Circos provides a number of example karyotype files for a number of model organisms; we now allow users to select one of these presets for their plot. This should make it easier for users to get started with the tool, by eliminating the need to provide a karyotype file themselves.

Given your observation that users familiar with Circos will be accustomed to providing bands in the karyotype file, combined with the addition of an option to select preset karyotype files (which include bands), we have made this the default way to specify bands, and removed the little-used option to provide a separate cytogenetic bands (BED) file. The tool will output the karyotype file to users upon request, to assist in the creation of their own custom karyotype files, by providing an example of the required file format.

2. Add type=highlight 2d track

> This track behaves exactly like a heatmap, except that it does not map the value of the data point onto a color. Instead, it uses the fill_color parameter.

Thanks! We had not previously seen this tab of the documentation.

Highlights were supported as a separate section (as block0. We took and merged that into the 2d type (type=highlight). So now in the wrapper and highlights will be data tracks with type=highlight, and users can use the z-index option to control plotting over or under other datasets as they like.

Does that sufficiently accomplish this? It seems sub-optimal from our perspective (maintenance) and from user experience to have this track type in two places with nearly identical behaviour except z-indexing? Or are there more differences that might necessitate us re-instating the proper highlight track type.

> The reason why this track is needed is because in the wrapper the heatmap track does not allow specifying a specific fill color for a data point (see below).

This should now be supported with the ‘advanced’ colour entry option.

3. Add z-index for plots and links

Previously this was only possible via rules, but we agree this is suboptimal and have now added a higher-level parameter to control this. We have fixed this in the latest version of the tool.

4. Add custom fields for plot and links

Unfortunately, as much as we would like to, we cannot currently address some parts of this point, due to the security risks involved in allowing user-provided free-text input in Galaxy.

We believe that if we provided a free-text field, we would not be able to sufficiently, safely sanitise this, preventing all malicious inputs. This is the reason why we have gone to significant lengths to make it possible to just use dropdowns and sliders to configure Circos, lest the user attempt supplying perl code which might be executed on the server.

If we would include such free-text inputs, the usegalaxy.* servers (and likely many others) would not be able to include the Circos tool due to the potential security implications. In the future, Galaxy is working towards more secure and isolated computation. Some servers have started implementing this, and when this has become the norm, we can update the Circos tool to allow such advanced options.

> There are a variety of settings for plots. Some very useful ones are
>
> minsize = MINSIZE

We have added this option since it sounds quite useful!

> Another is color_mapping for heatmaps

We have added this as well.

5. Fill color (especially for heatmaps)

We have implemented it as a text field. For the above-mentioned security reasons, this input is limited to strings matching [a-z0-9,-]+

6. Rules

> a. All actions (links/2d plots) should support the z-index (or z-depth as the wrapper calls it).

Done! And we have updated the text to z-index.

> b. There is a bug in how a rule interprets "change fill color based on value" when "0"

Fixed

> The wording "based on numerical position" is not intuitive and should be "based on value". Here, "position" can be easily confused with genomic position.

How about “Based on position on chromosome” which is the intention, and “based on position value” for the other? We have made this change, but if there is a better label, we can substitute that.

> The wording "expected minimum value of dataset" is not intuitive. It should be … "for points below value" … "for points above value"

We’ve changed these to just “minimum / maximum value” with a better explanation in the brewer scale selector:
Fill will be changed based on a mapping of dataset values to colours, but to do this the mapping needs to be defined. Minimum and maximum values are defined below, and will map to the minimum and maximum values of this brewer scale.

> The reason why this is very helpful is that you can combine several parameters in a data point
>
> hs1 10 20 0.5 myvar=0.75,myvar2=1.5,myvar3=2.5,…

Generating files like this is not trivial in Galaxy, so we had not encountered the need to support such a feature yet, most people just generate N data files using Galaxy collections and other features.

We have added support for this in case someone needs it.

7. Axes & Backgrounds

We recently removed it because some users found it confusing. We have re-added it and reworded it significantly, maybe it'll be clearer now.

8. Loading additional color profiles

This again would be untrusted user input, and would require significant sanitise rules to permit most valid human inputs. Given the additional comment of care needing to be taken to not redefine existing colours, implementing this would require significant work to ensure a good user experience. I am not sure it is possible to do this, and unfortunately we will have to relegate such a feature to being out of scope for the intention of the wrapper.

If we could identify some commonly used colour profiles we could easily build those into the tool?

9. Custom fields in each block

> Each block in the configuration file should have an option for a set of custom field definitions. This nicely gets around any functionality that the wrapper doesn't support.

see #4

> chromosomes_order

This is supported in the chromosome limit setting under Ideogram options. We decided to merge the limiting and ordering as a simplification for users.

This unfortunately comes again at the expense of limiting user terseness (if they want all the chromosomes with an order they just have to write it all), but at least in terms of Galaxy it should make the tool slightly more fool proof for untrained users.

We've updated the documentation to mention this additional ordering that is happening, so hopefully it is more clear.

10. Ideogram colors

> The "ideogram color scheme" currently isn't intuitive

This behaviour is specific to a couple of track types, and yes, misleading. We have reworked it, now the option is only visible for the tracks where it can be used (fasta to karyotype, lengths file to karyotype). And it won't be shown for a standard karyotype input where the colours won't be overridden. Additionally when the user uses a fasta or length file as input, we now output the karyotype file so they can re-use this karyotype file directly rather than having that be re-calculated every time. Hopefully this will get non-Circos familiar users more experience with Circos' karyotype files.

> The wrapper behaves strangely when not auto-detecting a file as BED. If I upload a .txt file (e.g. space-delimited) and not explicitly convert it to BED then it doesn't show up in the file dropdown list. When using the folder icon next to the dropdown list the file is prefixed with "(unavailable)".

Ah there are two folder icons, on the left is the "Collection input" (not relevant here), on the right is the new dataset selector. In this case, it cannot auto-convert the datatypes I guess, so, it refuses to accept

this input.

Important

> I don't think it's important to insist on BED (which is a loose format anyway).
> When viewing the data file in galaxy, the entire line shows up in the first column.

For us, one of Galaxy’s main selling points is that users cannot provide really wrong inputs to a tool, e.g. you cannot select a PNG file where a table input is required. Likewise, you cannot provide a generic table, when we know that the tool needs an Interval format file (bed3 + any number of columns after).

By enforcing the permitted input file types we help ensure our users cannot provide wrong inputs and remove a class of potential support issues we would have to solve on a regular basis.

Additionally, given that numerous other Galaxy tools exist to process tabular data (cutting rows and columns, computing expressions on columns, etc.), and they all have a hard requirement on tab-separated data, we fear we would be hurting the user’s potential Galaxy experience, if we permitted .txt files, and they could only send them to Circos, and were unable to use them in many tools or pipelines that might exist on those servers.

Space separated data can be converted on upload with the built in option of converting spaces to tabs (under gear icon) if this helps?

TODO

> Add connector track (2d track type).

Done!

Additionally we have implemented the “Zoom” feature since we stumbled across it in the documentation and it looked very useful.

Reviewer #2

Thanks a lot for your valuable comments and insights. We have updated the manuscript according to your comments. More detailed point-by-point responses are given below.

> Choose to use either "visualisation" or "visualization"

We have replaced all british-isms with american english for consistency.

> Consider reformatting manuscript title to aid with searches (yes, this is more formal!): "Galactic Circos: User-friendly Circos Plots within the Galaxy platform".

Done! Thanks for the tip.

> Consider the use of upper case "X" and "Y" chromosome nomenclature in Figure 4 (and in the underlying input data at Zenoto, if possible):

That side of the image was the original image appearing in a previous publication which we were reproducing with our tool, so we cannot change that part of the image unfortunately. However, we do agree with you that uppercase should be the default. As part of this revision, we added a set of preconfigured karyotype files for model organisms to the tool, so that users who use one of these model organisms do not have to supply a karyotype file. In these presets, the X and Y chromosomes have uppercase labels, so this should be the default behaviour of the tool now, unless users manually supply a karyotype file with lowercase chromosome labels.

> Consider alternating formal and informal variations of verbiage: The term "tweak" may be a bit overused

Thanks, we have reduced the use of the word tweaking, and hope it is more aligned with Circos nomenclature now.

> Consider consistent naming when referencing the Galaxy ToolShed:

Done!

> Consider adding in links and resources for all items listed under "Availability of source code and requirements".

Great idea, we have added your suggested links (and a few additional ones).

Reviewer #3

Thank you for your review, we have addressed the concerns you raised. We have added a section describing the lessons learned and listing more explicitly the limitations of the tool. We have also updated the sharing settings for the inaccessible history link. Regarding the figures, we have high-quality source images for all figures, and will confer with the editors about the best placement of these, since the current configuration was dictated by the LaTeX template, but may be altered for the final publication.

> To the best of my knowledge, this is one of the more comprehensive efforts to integrate a visualization package into Galaxy or similar GUI workflow platform. Given the unique aspect of this work, it would useful for the manuscript to have a section discussing lessons learned as well as limitations of the tool.

We have added a section describing numerous lessons learnt in the paper, attempting to filter out those which would be useful for an average reader. There are numerous additional issues or pain points which could be expounded upon but would be too technical in this context. We can open discussions with the Galaxy team regarding how those might be fixed.

Close
