# Supplementary material for: Galactic Circos: User-friendly Circos plots within the Galaxy platform
Source: Gigascience. 2020 Jun 12;9(6):giaa065. doi: 10.1093/gigascience/giaa065 (PMC7291503; doi:10.1093/gigascience/giaa065)
Supplement: giaa065_Response_to_Reviewer_Comments_Revision_1 [file giaa065_response_to_reviewer_comments_revision_1.pdf]

Author's Response To Reviewer Comments

Close

> I see you've added the GigaDB reference, but can you actually cite it from the "Availability of supporting data and materials" section instead?

Sure, of course! Done.

> Then you need to cite the DOI rather than the URL as the URL may change when we update the database. So this reference needs to be:

No problem, I've updated the URL field in the references accordingly.

I've added DOIs to a few of the other references which had been missing them before as well, thank you for the reminder.

> As a final change it would be useful if you put both your ORCID IDs by your names so they can be linked.

Done, thank you for using ORCIDs! I hope I have done them in the correct format, I didn't find any guidelines for this portion but maybe I didn't look in the right places.

Thank you for all of the time helping us prepare this submisison!

Close
