# Supplementary material for: Galactic Circos: User-friendly Circos plots within the Galaxy platform
Source: Gigascience. 2020 Jun 12;9(6):giaa065. doi: 10.1093/gigascience/giaa065 (PMC7291503; doi:10.1093/gigascience/giaa065)
Supplement: giaa065_Reviewer_1_Report_Original_Submission — Martin Krzywinski, M.Sc. -- 2/20/2020 Reviewed [file giaa065_reviewer_1_report_original_submission.pdf]

**Reviewer Report**

**Title: Galactic Circos: User-friendly Circos Plots within the Galaxy platform**

**Version: Original Submission** **Date:** 2/20/2020

**Reviewer name: Martin Krzywinski, M.Sc.**

**Reviewer Comments to Author:**

The Circos Galaxy wrapper is a much-needed tool and is sure to receive a warm welcome by the community.
The paper is soundly written. I suggest more emphasis be placed on the flexible support for rules--the fact that the way data is displayed can be altered based on position, data values or other parameters that are meaningful to the task at hand without adjusting input data files.
I've had the opportunity to use the wrapper for several days while training users of Galaxy Australia.
https://usegalaxy.org.au/u/martin-krzywinski/h/circos-workshop-v2-australia-biocommons
During that time, I found a few incongruencies, bugs and missing features that need to be addressed before publication. I've categorized these below as "critical" and others as "important" and "todo".
Some of these may have already been addressed in the latest patch.
CRITICAL
1. Karyotype bands
Conventionally (by this I mean in Circos), cytogenetic band definitions are included in the karyotype file.
However, they are not drawn by the wrapper unless I separately define these in the (Ideogram > Cytogenetic bands) section.
There should be a toggle to show bands, which sets show_bands in the block.
...
show_bands = yes
The option to "convert bands from BED to Circos format" is confusing and by default set to "yes". It's confusing because if the original Circos band format is used here, it's normally included in the karyotype.
It would be better to not mention "Circos karyotype band format" at all and instead accept an default data track format like this
CHR START END 0 fill_color=gneg
...
2. Add type=highlight 2d track
This is a very important track type which should be included. See
http://www.circos.ca/documentation/tutorials/highlights/on_data/configuration
This track behaves exactly like a heatmap, except that it does not map the value of the data point onto a color. Instead, it uses the fill_color parameter.
The reason why this track is needed is because in the wrapper the heatmap track does not allow specifying a specific fill color for a data point (see below).
type = highlight
fill_color = black

...

3. Add z-index for plots and links

Currently, plots are drawn in the order they are defined in the wrapper. There is no way to rearrange their order.

Circos handles this by assigning a z value to each and block. By default z = 0 but may be locally changed

...

z = 10

to draw the plot after all those with z < 10. This parameter should be included in each form, probably after radii definition and before "plot type" definition.

4. Add custom fields for plot and links

There are a variety of settings for plots. Some very useful ones are

minsize = MINSIZE

which enlarges the data point to be of size MINSIZE.

Another is color_mapping for heatmaps, see

http://www.circos.ca/documentation/tutorials/2d_tracks/heat_maps/

I suggest that there be a text box that allows me to append these custom definitions (here the user should be warned this is an advanced feature).

Possibly after "backgrounds". The user would paste in

---

min_size = 10u

---

and this would be inserted into the corresponding or block.

Note that the user can suffix the parameter with "*" to overwrite any other definition of that parameter in the block. Thus, they can paste

---

min_size = 10u

fill_color* = red

---

and any fill_color defined by the wrapper would be redefined to red. In other words,

...

fill_color = green

fill_color* = red

would use the red value. The reason why "*" is required is that the Circos file parser treats multiple definitions as a list, which is sometimes what you actually want.

5. Fill color (especially for heatmaps)

The fill color option should be available as a color picker (as it is currently) but also as a text field in which I can specify the color name. The benefit of this is being able to select individual Brewer colors directly (reds-5-seq-3).

More importantly, though, this very much applies to how color is defined for heatmaps. Circos defines the color of a heatmap as a string which can be either a color or a color list. For example

color = red # single color for all points

color = reds-5-seq # 5-color Brewer palette

But color can also be defined as a CSV which generates a user-defined color ramp

color = red,blue # first 50% points red, rest blue

color = blues-5-seq-rev,reds-5-seq

Two color lists combined into a bigger list.

By providing a text field to define color, all these are seamlessly available.

6. Rules

Rules are largely well implemented. But a few tweaks are needed - I noticed that different track types have different actions that are available. These should be standardized as much as possible.

a. All actions (links/2d plots) should support the z-index (or z-depth as the wrapper calls it).

b. There is a bug in how a rule interprets "change fill color based on value" when "0" is used for either the minimum or maximum as well as for the condition "based on numerical position". For example, for the latter the config file would have

condition = var(value) >0

...

but something goes wrong with the wrapper (I suspect there's an if statement which evaluates false on the "0") and the Circos instance crashes with the following error (Galaxy Australia toolshed.g2.bx.psu.edu/repos/iuc/circos/circos/0.69.8+galaxy4.)

```
at /mnt/pulsar/dependencies/_conda/envs/mulled-v1-dd38f3b35796eaa94d0b5d1d9c1428ec987dcd7f39e81a08df8df138203a8004/bin/../lib/Circos/Error.pm line 425, line 5515.
    Circos::Error::fatal_error("rule", "no_condition_no_flow", "{\x{a}  'fill_color' => '255, 0, 0'\x{a}}\x{a}") called at /mnt/pulsar/dependencies/_conda/envs/mulled-v1-dd38f3b35796eaa94d0b5d1d9c1428ec987dcd7f39e81a08df8df138203a8004/bin/../lib/Circos/Rule.pm line 80
    Circos::Rule::make_rule_list(ARRAY(0x55d1ae1b9050)) called at /mnt/pulsar/dependencies/_conda/envs/mulled-v1-dd38f3b35796eaa94d0b5d1d9c1428ec987dcd7f39e81a08df8df138203a8004/bin/../lib/Circos.pm line 1483
    Circos::run("Circos", "_argv", "-conf circos/conf/circos.conf -noparanoid", "paranoid", 0, "configfile", "circos/conf/circos.conf", "_cwd", ...) called at /mnt/pulsar/dependencies/_conda/envs/mulled-v1-dd38f3b35796eaa94d0b5d1d9c1428ec987dcd7f39e81a08df8df138203a8004/bin/circos line 536
```

The wording "based on numerical position" is not intuitive and should be "based on value". Here, "position" can be easily confused with genomic position.

The wording "expected minimum value of dataset" is not intuitive. It should be

"for points below value"

[ value ]

"for points above value"

[ value]

There should also be an action that allows for a custom variable=value assignment. This would allow someone to write a rule like this

condition = 1

value = var(myvar)

for a data point that had myvar as a parameter

hs1 10 20 0.5 myvar=0.75

The reason why this is very helpful is that you can combine several parameters in a data point

hs1 10 20 0.5 myvar=0.75,myvar2=1.5,myvar3=2.5,...

and then use them as values for a plot without having to create a new file.

This also allows coloring data (e.g. links) by the color of the chromosome

condition = 1

color = eval(var(chr1))

7. Axes & Backgrounds

Should support absolute and relative start/end positions.

http://www.circos.ca/documentation/tutorials/quick_start/axes_and_backgrounds/

This can be done by just appending "r" to the start/end/spacing

y0 = 0

y1 = 0.5

spacing = 0.1

y0 = 0.25r

y1 = 0.75r

spacing = 0.1r

The same applies for backgrounds, which actually benefit from this more. Currently, only relative background positions are supported. Absolute positions are needed for cases where the user wants to highlight values larger/smaller than a certain value, e.g.

min = 0

max = 10

y0 = 8

color = red

Note that when y1 is not defined it is assumed to be "1r" and when y0 isn't defined it is assumed to be "0r".

8. Loading additional color profiles

User should be able to upload their own color definitions which are inserted into the block. Care would need to be taken that the user doesn't redefine existing colors.

To overwrite color names use "*", see

http://circos.ca/documentation/tutorials/quick_start/links_and_rules/

9. Custom fields in each block

Each block in the configuration file should have an option for a set of custom field definitions. This nicely gets around any functionality that the wrapper doesn't support. For example

chromosomes_order

and

chromosomes_color

see

http://circos.ca/documentation/tutorials/quick_start/links_and_rules/

10. Ideogram colors

The "ideogram color scheme" currently isn't intuitive. If you upload a karyotype file with the

chromosome color defined (e.g. default human karyotype that ships with Circos) then the ideograms are colored by this definition as opposed to the color scheme defined in the wrapper.
IMPORTANT
The wrapper behaves strangely when not auto-detecting a file as BED. If I upload a .txt file (e.g. space-delimited) and not explicitly convert it to BED then it doesn't show up in the file dropdown list. When using the folder icon next to the dropdown list the file is prefixed with "(unavailable)".
I don't think it's important to insist on BED (which is a loose format anyway).
When viewing the data file in galaxy, the entire line shows up in the first column.
TODO
Add connector track (2d track type).
http://circos.ca/documentation/tutorials/2d_tracks/connectors/

**Level of Interest**

Please indicate how interesting you found the manuscript: Choose an item.

**Quality of Written English**

Please indicate the quality of language in the manuscript: Choose an item.

**Declaration of Competing Interests**

Please complete a declaration of competing interests, considering the following questions:

- Have you in the past five years received reimbursements, fees, funding, or salary from an organisation that may in any way gain or lose financially from the publication of this manuscript, either now or in the future?
- Do you hold any stocks or shares in an organisation that may in any way gain or lose financially from the publication of this manuscript, either now or in the future?
- Do you hold or are you currently applying for any patents relating to the content of the manuscript?
- Have you received reimbursements, fees, funding, or salary from an organization that holds or has applied for patents relating to the content of the manuscript?
- Do you have any other financial competing interests?
- Do you have any non-financial competing interests in relation to this paper?

If you can answer no to all of the above, write 'I declare that I have no competing interests' below. If your reply is yes to any, please give details below.

I declare that I have no competing interests

I agree to the open peer review policy of the journal. I understand that my name will be included on my report to the authors and, if the manuscript is accepted for publication, my named report including any attachments I upload will be posted on the website along with the authors' responses. I agree for my report to be made available under an Open Access Creative Commons CC-BY license (http://creativecommons.org/licenses/by/4.0/). I understand that any comments which I do not wish to be included in my named report can be included as confidential comments to the editors, which will not be published.

Choose an item.

To further support our reviewers, we have joined with Publons, where you can gain additional credit to further highlight your hard work (see: https://publons.com/journal/530/gigascience). On publication of this paper, your review will be automatically added to Publons, you can then choose whether or not to claim your Publons credit. I understand this statement.

Yes Choose an item.
