# Supplementary material for: Galactic Circos: User-friendly Circos plots within the Galaxy platform
Source: Gigascience. 2020 Jun 12;9(6):giaa065. doi: 10.1093/gigascience/giaa065 (PMC7291503; doi:10.1093/gigascience/giaa065)
Supplement: giaa065_Reviewer_1_Report_Revision_1 — Martin Krzywinski, M.Sc. -- 5/11/2020 Reviewed [file giaa065_reviewer_1_report_revision_1.pdf]

Reviewer Report

Title: Galactic Circos: User-friendly Circos Plots within the Galaxy platform

Version: Revision 1 **Date:** 5/11/2020

Reviewer name: Martin Krzywinski, M.Sc.

Reviewer Comments to Author:

Reviewer #1
2. Add type=highlight 2d track
>> This track behaves exactly like a heatmap, except that it does not map the value of the data point onto a color. Instead, it uses the fill_color parameter.
>Thanks! We had not previously seen this tab of the documentation.
>Highlights were supported as a separate section (as block0. We took and merged that into the 2d type (type=highlight). So now in the wrapper and highlights will be data tracks with type=highlight, and users can use the z-index option to control plotting over or under other datasets as they like.
The "highlight" track via is pretty much deprecated in favour of with type=highlight.
To streamline the UI, remove.
4. Add custom fields for plot and links
> Unfortunately, as much as we would like to, we cannot currently address some parts of this point, due to the security risks involved in allowing user-provided free-text input in Galaxy.
Understood. Perhaps in the future custom fields could be composed from a set of validated keywords.
>> The wording "based on numerical position" is not intuitive and should be "based on value". Here, "position" can be easily confused with genomic position.
>How about "Based on position on chromosome" which is the intention, and "based on position value" for the other? We have made this change, but if there is a better label, we can substitute that.
I would use "position" for anything related to start/end on the axis and "value" for the data point's value. "Position on chromosome" works as well, but "position value" mixes up the two and it's no longer clear whether we're dealing with the "value of position" or "value at position".
> The reason why this is very helpful is that you can combine several parameters in a data point
>
> hs1 10 20 0.5 myvar=0.75,myvar2=1.5,myvar3=2.5,…
>Generating files like this is not trivial in Galaxy, so we had not encountered the need to support such a feature yet, most people just generate N data files using Galaxy collections and other features.
>We have added support for this in case someone needs it.
Great. Some fraction of users might be creating their files out-of-band and then popping into Galaxy with them ready.
>8. Loading additional color profiles
>This again would be untrusted user input, and would require significant sanitise rules to permit most valid human inputs. Given the additional comment of care needing to be taken to not redefine existing colours, implementing this would require significant work to ensure a good user experience. I am not

sure it is possible to do this, and unfortunately we will have to relegate such a feature to being out of scope for the intention of the wrapper.
>If we could identify some commonly used colour profiles we could easily build those into the tool?
Yes, you can create collections of colors in a list and then allow users to include these via some kind of check box scheme. As long as the brewer colors are available, as well as RGB and HSV, then it should be fine.
>10. Ideogram colors
> The "ideogram color scheme" currently isn't intuitive
>This behaviour is specific to a couple of track types, and yes, misleading. We have reworked it, now the option is only visible for the tracks where it can be used (fasta to karyotype, lengths file to karyotype). And it won't be shown for a standard karyotype input where the colours won't be overridden. Additionally when the user uses a fasta or length file as input, we now output the karyotype file so they can re-use this karyotype file directly rather than having that be re-calculated every time. Hopefully this will get non-Circos familiar users more experience with Circos' karyotype files.
If you're generating karyotypes from user input, it's safest to not color the segements intially and set their values to "black". For anything more than 10-12 chromosomes, you'll quickly run out of Brewer colors or any other non-continuous color palette. One option is to assign the colors based on LCH color values (Circos supports lch(l,c,h) as a color value) and then let the user specify chroma (C) and luminance (L) and you would cycle H over the chromosomes. This would give a more-or-less brigthness/saturation normalized rainbow.
If you generate this color file for the user (e.g. chr1color = ..., chr2color = ...) then they can use these colors by name to color the data.

Level of Interest

Please indicate how interesting you found the manuscript: Choose an item.

Quality of Written English

Please indicate the quality of language in the manuscript: Choose an item.

Declaration of Competing Interests

Please complete a declaration of competing interests, considering the following questions:

- Have you in the past five years received reimbursements, fees, funding, or salary from an organisation that may in any way gain or lose financially from the publication of this manuscript, either now or in the future?
- Do you hold any stocks or shares in an organisation that may in any way gain or lose financially from the publication of this manuscript, either now or in the future?

- Do you hold or are you currently applying for any patents relating to the content of the manuscript?
- Have you received reimbursements, fees, funding, or salary from an organization that holds or has applied for patents relating to the content of the manuscript?
- Do you have any other financial competing interests?
- Do you have any non-financial competing interests in relation to this paper?

If you can answer no to all of the above, write 'I declare that I have no competing interests' below. If your reply is yes to any, please give details below.

I declare that I have no competing interests.

I agree to the open peer review policy of the journal. I understand that my name will be included on my report to the authors and, if the manuscript is accepted for publication, my named report including any attachments I upload will be posted on the website along with the authors' responses. I agree for my report to be made available under an Open Access Creative Commons CC-BY license (http://creativecommons.org/licenses/by/4.0/). I understand that any comments which I do not wish to be included in my named report can be included as confidential comments to the editors, which will not be published.

Choose an item.

To further support our reviewers, we have joined with Publons, where you can gain additional credit to further highlight your hard work (see: https://publons.com/journal/530/gigascience). On publication of this paper, your review will be automatically added to Publons, you can then choose whether or not to claim your Publons credit. I understand this statement.

Yes Choose an item.
