# Supplementary material for: Galactic Circos: User-friendly Circos plots within the Galaxy platform
Source: Gigascience. 2020 Jun 12;9(6):giaa065. doi: 10.1093/gigascience/giaa065 (PMC7291503; doi:10.1093/gigascience/giaa065)
Supplement: giaa065_Reviewer_2_Report_Original_Submission — Jennifer Hillman-Jackson -- 2/25/2020 Reviewed [file giaa065_reviewer_2_report_original_submission.pdf]

Reviewer Report

Title: Galactic Circos: User-friendly Circos Plots within the Galaxy platform

Version: Original Submission **Date:** 2/25/2020

Reviewer name: Jennifer Hillman-Jackson

Reviewer Comments to Author:

Suggested (minor/optional) language edits to enhance clarity and overall context.
â€¨
Consider small modifications to the abstract that help to orient readers not yet familiar with the Galaxy platform, the Circos tool itself, and the concept of what a "tool wrapper" is:
â€¨
Background: Circos is a popular software package for the circular visualisation of complex datasets. Circos is a highly flexible tool. While especially popular in the field of genomic analysis, Circos enables interactive graphing of any analytical data, including alternative scientific domain data and non-scientific data. This high degree of flexibility also comes with a high degree of complexity, which may present an obstacle for researchers not
trained in programming or the UNIX command line. The Galaxy platform provides a user-friendly graphical interface incorporating a broad range of "wrapped" command-line tools to facilitate accessibility.
Findings: We have developed a Galaxy wrapper for Circos, thus combining the power of Circos with the accessibility and ease-of-use of the Galaxy platform. The combination significantly simplifies the specification and configuration of Circos plots for end-users while
retaining the power to produce publication-quality visualizations* of complex multidimensional datasets.
Conclusions: Galactic Circos enables the creation of publication-ready Circos plots within the Galaxy platform. Users may download the full configuration of their plots for further manual tweaking. This version of Circos is available as an open-source installable application from the Galaxy ToolShed, with its usage clarified in a training manual hosted by the Galaxy Training Network.
â€¨
Consider standardizing vocabulary terms where alternative spelling is possible and correct (throughout the document):
Choose to use either "visualisation" or "visualization" consistently throughout the document. The Galaxy platform itself uses "visualize" and "visualization" in the GUI navigation labels, and it may be less confusing for end-users to also use that form here.
â€¨
Consider reformatting manuscript title to aid with searches (yes, this is more formal!):
"Galactic Circos: User-friendly Circos Plots within the Galaxy platform".
â€¨
Consider the use of upper case "X" and "Y" chromosome nomenclature in Figure 4 (and in the underlying

input data at Zenoto, if possible):
plot-complete-genomics-both.png
â€¨
Consider alternating formal and informal variations of verbiage:
The term "tweak" may be a bit overused and loses impact upon repetition. "Configuration", "Options", "Parameters" are just some alternatives. Using nomenclature that mirrors that of the original Circos authors (in their manual/publication) will make it easier for end-users to utilize those resources when exploring the application as wrapped for Galaxy.
Consider consistent naming when referencing the Galaxy ToolShed:
"ToolShed" is one word in publications from the Galaxy team. It is suggested to use the same nomenclature in this manuscript as well.
Consider adding in links and resources for all items listed under "Availability of source code and requirements". In short, assume the reader is starting from scratch, needs context, and doesn't understand where to find existing resources or real-time assistance. Examples:
Where to get Galaxy: Repositories, Docker, CloudLaunch … URLs
How to install Galaxy: https://getgalaxy.org
How to install tools: Tutorials, admin docs and training
Full Administrative resources: https://docs.galaxyproject.org/
Help/Support (end-user and admin): Gitter chats, Galaxy Help
Public Galaxy servers that include Circos: usegalaxy.* servers?

Level of Interest

Please indicate how interesting you found the manuscript: Choose an item.

Quality of Written English

Please indicate the quality of language in the manuscript: Choose an item.

Declaration of Competing Interests

Please complete a declaration of competing interests, considering the following questions:

- Have you in the past five years received reimbursements, fees, funding, or salary from an organisation that may in any way gain or lose financially from the publication of this manuscript, either now or in the future?
- Do you hold any stocks or shares in an organisation that may in any way gain or lose financially from the publication of this manuscript, either now or in the future?
- Do you hold or are you currently applying for any patents relating to the content of the manuscript?
- Have you received reimbursements, fees, funding, or salary from an organization that holds or has applied for patents relating to the content of the manuscript?

- Do you have any other financial competing interests?
- Do you have any non-financial competing interests in relation to this paper?

If you can answer no to all of the above, write 'I declare that I have no competing interests' below. If your reply is yes to any, please give details below.

Member of the Galaxy Team. Galaxy is an fully open-source platform. I declare that I have no competing interests.

I agree to the open peer review policy of the journal. I understand that my name will be included on my report to the authors and, if the manuscript is accepted for publication, my named report including any attachments I upload will be posted on the website along with the authors' responses. I agree for my report to be made available under an Open Access Creative Commons CC-BY license (http://creativecommons.org/licenses/by/4.0/). I understand that any comments which I do not wish to be included in my named report can be included as confidential comments to the editors, which will not be published.

Choose an item.

To further support our reviewers, we have joined with Publons, where you can gain additional credit to further highlight your hard work (see: https://publons.com/journal/530/gigascience). On publication of this paper, your review will be automatically added to Publons, you can then choose whether or not to claim your Publons credit. I understand this statement.

Yes Choose an item.
