# Supplementary material for: Galactic Circos: User-friendly Circos plots within the Galaxy platform
Source: Gigascience. 2020 Jun 12;9(6):giaa065. doi: 10.1093/gigascience/giaa065 (PMC7291503; doi:10.1093/gigascience/giaa065)
Supplement: giaa065_Reviewer_3_Report_Original_Submission — Jeremy Goecks -- 2/26/2020 Reviewed [file giaa065_reviewer_3_report_original_submission.pdf]

Reviewer Report

Title: Galactic Circos: User-friendly Circos Plots within the Galaxy platform

Version: Original Submission **Date:** 2/26/2020

Reviewer name: Jeremy Goecks

Reviewer Comments to Author:

The authors describe a Galaxy tool for generating Circos plots. The tool provides most of the functionality of the underlying Circos plotting package, and additional Galaxy tools are available to convert data into formats that can be used with the Galaxy tool. This work is well motivated, leveraging the popular Galaxy platform to make the widely-used Circos tool more readily available. Methods and results are clearly described, the manuscript is well written, and there are web links to reproduce the work in the paper plus tutorials on how to use the tool. Overall this is a very nice technical note, and the work described is important and relevant.
Major issues:
- None
Minor issues:
- To the best of my knowledge, this is one of the more comprehensive efforts to integrate a visualization package into Galaxy or similar GUI workflow platform. Given the unique aspect of this work, it would useful for the manuscript to have a section discussing lessons learned as well as limitations of the tool. These are mentioned in the text in various places but collecting them in one place would be very valuable for readers. Adding this section at the end of the results would be great.
- https://usegalaxy.eu/u/saskia/h/circos-politics-plot is not accessible to me
- Circos figures are small and difficult to appreciate. Perhaps remove a figure or even two so that other figures can be made larger.

Level of Interest

Please indicate how interesting you found the manuscript: Choose an item.

Quality of Written English

Please indicate the quality of language in the manuscript: Choose an item.

Declaration of Competing Interests

Please complete a declaration of competing interests, considering the following questions:

- Have you in the past five years received reimbursements, fees, funding, or salary from an organisation that may in any way gain or lose financially from the publication of this manuscript, either now or in the future?

- Do you hold any stocks or shares in an organisation that may in any way gain or lose financially from the publication of this manuscript, either now or in the future?
- Do you hold or are you currently applying for any patents relating to the content of the manuscript?
- Have you received reimbursements, fees, funding, or salary from an organization that holds or has applied for patents relating to the content of the manuscript?
- Do you have any other financial competing interests?
- Do you have any non-financial competing interests in relation to this paper?

If you can answer no to all of the above, write 'I declare that I have no competing interests' below. If your reply is yes to any, please give details below.

Yes. J Goecks has a significant financial interest in Galactic Core, a company that may have a commercial interest in the results described in this manuscript. This potential conflict of interest has been reviewed and managed by OHSU.

I agree to the open peer review policy of the journal. I understand that my name will be included on my report to the authors and, if the manuscript is accepted for publication, my named report including any attachments I upload will be posted on the website along with the authors' responses. I agree for my report to be made available under an Open Access Creative Commons CC-BY license (http://creativecommons.org/licenses/by/4.0/). I understand that any comments which I do not wish to be included in my named report can be included as confidential comments to the editors, which will not be published.

Choose an item.

To further support our reviewers, we have joined with Publons, where you can gain additional credit to further highlight your hard work (see: https://publons.com/journal/530/gigascience). On publication of this paper, your review will be automatically added to Publons, you can then choose whether or not to claim your Publons credit. I understand this statement.

Yes Choose an item.
